# A cloud server centric multifactor lightweight authentication scheme for eHealth systems

**DOI:** 10.1038/s41598-026-40356-2

**Published:** 2026-03-14

**Authors:** Divyanshu Gairola, Pramod Kumar Maurya, Ankush Chanda

**Affiliations:** 1 Department of Mathematics, School of Advanced Sciences, Vellore Institute of Technology, Vellore, Tamil Nadu India; 2 BML Munjal University, Gurugram, India

**Keywords:** Authentication protocol, E-health systems, IoT, TMIS, Wireless communication, Computational biology and bioinformatics, Engineering, Health care, Mathematics and computing

## Abstract

The safety of transmitted data is an essential element of all Cloud-IoT-based electronic healthcare (e-healthcare) systems. Through a review of prior research, we see that there have been several different security frameworks proposed for the protection of communications between the patient, the provider and the cloud server, but most of them have significant weaknesses related to serious attack vectors such as man-in-the-middle, impersonation and denial-of-service attacks. The existence of these vulnerabilities places the sensitive health care information at risk of being compromised in terms of confidentiality and integrity. In recent years, Alzahrani et al. provided a provably secure cloud-centric authentication protocol for use with e-health care systems. It was found through a detailed analysis that their protocol lacked the robust authentication that is required to protect the system from impersonation attacks by attackers on the cloud server or the physician. Therefore, in order to resolve the issues associated with the lack of robustness, this paper provides a cloud-server-centric multi-factor authentication protocol for use in the health care environment. This protocol has the ability to combine the features of one-way hash functions, biometric identification and random number generation to provide increased security in the process of authenticating users to access the e-health care system while mitigating the previously identified vulnerabilities. The correctness and robustness of the protocol were formally analyzed using BAN Logic, the Real-Or-Random Security Model, formal verification using AVISPA Tool and practical implementation analysis. Additionally, the performance of the protocol was measured in terms of computational time, communication overhead and scalability. The results of the security analysis indicate that the proposed protocol can withstand all types of attacks on e-health care systems. Furthermore, the performance analysis demonstrated that the protocol achieved improved efficiencies over the current state-of-the-art protocols in each of the measured performance characteristics. As a result, the proposed protocol has the potential to be used in practice as part of cloud-IoT-based e-healthcare applications.

## Introduction

Cloud computing is used in many areas, including logistics, big data analytics, marine safety, and especially e-healthcare systems, where it is an important part of making services better, easier to get to, and more efficient. This is because it can be changed and made bigger to better meet different needs than traditional physical infrastructure. We can use the paradigm in many different ways, like in private, public, hybrid, and multi-cloud environments. Public clouds are hosted by third-party companies and are open to anyone on the Internet. On the other hand, private clouds are only for one business and can be hosted by that business or by another company. Hybrid solutions combine public and private clouds to give businesses more options. Multi-cloud solutions, on the other hand, use services from more than one source to make systems more stable, save money, and work better by not depending on just one vendor. Despite the widespread usage of cloud computing in e-healthcare systems, secure data transfer remains a major concern. Unauthorised access to a patient’s medical records might have serious consequences. Other concerns include the integrity and accuracy of data, the authentication and authorisation of patients, doctors, and servers, the security of devices and networks, and the secure transmission of data.

The main idea behind this model is that it lets people access computing power, storage, and software applications on demand. This means that businesses don’t have to keep their own infrastructure or buy special hardware. Cloud service providers (CSPs) are in charge of managing and maintaining the underlying infrastructure. This lets users focus on using the services effectively instead of managing the hardware. Cloud computing is a big change in how computing resources are given out and used over the Internet.

The use of cloud computing in telecare medical information systems (TMIS)^1^ offers resources like data storage, processing power, and software. This development drives progress. It helps medical professionals boost their productivity, improve collaboration, and provide better patient diagnosis and care. The reliability of these systems is further enhanced by wearable technology, sensors, and IoT technologies. These tools connect with cloud servers, enabling remote monitoring of a vulnerable patient’s data and vital signs. Additionally, cloud-based video conferencing strengthens e-healthcare services. It allows doctors to consult with patients securely from a distance and work effectively with other medical professionals.

Beyond connectivity, cloud computing helps healthcare organizations handle large amounts of medical data. It allows them to find patterns and gain valuable insights that support clinical decision-making and improve patient outcomes. Providers can use cloud platforms to apply artificial intelligence and machine learning models. This enables applications like infection detection, outcome prediction, and personalized treatment planning^2^. Given these benefits, e-healthcare systems with cloud support combine different technologies that physicians and patients use. This approach reduces the need for costly physical infrastructure and allows organizations to adjust computational resources easily. It reduces costs and simplifies sharing workloads among stakeholders. This, in turn, helps with resource management and makes operations more efficient.

However, the overall security of patient records at all entities depends on the reliability of service providers, the strength of their security protocols, and strict compliance with regulations. These measures are necessary to protect against cyber threats, unauthorized access, and system weaknesses.

### Motivation

Cloud-based health care requires the protection of individual privacy because patient health care records and other confidential information are typically accessed many times per day. Due to increased use of cloud technology, these issues have made it easier for hackers to gain unauthorized access to your personal data. Because of the large volume and complexity of health care data, traditional authentication technologies do not provide sufficient security in cloud computing environments. Many of today’s authentication technologies will sacrifice one aspect of user experience (usability) in order to enhance another aspect (security). The added computational load associated with additional security features will further reduce the ability to scale or perform cloud-based health care applications. As a result of the competing demands of security, privacy, and usability, current authentication technologies have not provided an adequate solution for the protection of personal health care data.

### Main contribution

- Alzahrani et al.^3^ introduced an authentication protocol for the cloud-based healthcare system. We have shown that the proposed authentication scheme does not provide sufficient protection from attacks on impersonation that target the cloud server and the physician. Because of these vulnerabilities, we are developing an enhanced authentication scheme for cloud-based health care systems.
- We develop a new cloud-based IoT (Internet of Things) based multi-factor authentication protocol for e-Healthcare Systems, called CSMAE. CSMAE consists of three main entities: (1) doctor/physician (or medical staff), (2) Cloud Server, (3) Patients/Sensor Groups. In CSMAE, both physicians/users and patients/sensors register with the Cloud Server. Following this registration process, the two parties perform a mutual authentication and generate a secure session key for confidentiality and reliability of communication between the two parties.
- CSMAE employs multi-factor authentication for all doctors/users through the combination of three factors: knowledge (password), inherence (biometric), and possession (smart-card). Therefore, only authorized users will be able to obtain sensitive patient data.
- In order to be computationally efficient, the new protocol uses only the most elementary arithmetic/bitwise operations and a simple cryptographic hashing function. It does not make use of elliptical curve point multiplications, and therefore it does not use standard symmetric or asymmetric encryption methods; as such, it has significantly less processing power requirements than do other current protocols.
- The protocol also includes useful features for maintaining a working system, e.g., ownership transfer, user/doctor’s password change, etc.; this will enhance its usability/operability in actual clinical settings.
- The performance of our protocol is measured by communication cost, computation cost, energy cost and scalability; these measurements show that CSMAE outperforms representative “state of the art” protocols.
- The security of CSMAE is demonstrated using both formal and informal techniques. Formal analysis will include BAN logic, the Real-or-Random (ROR) model and AVISPA. Informal analysis will include an attack-based approach, along with illustrative scenarios demonstrating that CSMAE can resist all known attacks against the adversary model being used.

### Paper organisation

The remainder of this paper is organised as follows. Section “Literature survey” reviews the related literature in both textual and tabular form. Section “Preliminaries” introduces the necessary preliminaries and notations. An overview and cryptanalysis of Alzahrani et al.’s protocol are discussed in Section "Overview of Alzahrani et al.’s protocol". In Section “Proposed work”, the proposed protocol is presented. The security analysis of the scheme is detailed in Section “Security analysis”, while Section “Performance analysis” discusses the performance evaluation. Finally, Section “Conclusion” concludes the paper with closing remarks.

## Literature survey

In order to provide a viable and reliable platform for the deployment of cloud-integrated e-healthcare systems, the development of secure and dependable authentication mechanisms is required. Secure authentication is vital for ensuring the confidentiality and integrity of sensitive information stored in these applications including that of the patient. As a result of this, there has been much research into developing authentication protocols specifically suited to these types of systems. Examples include the work of Lopes et al.^4^, who created a protocol for enabling both secure and mutual authentication between different devices in an e-health environment. The drawback of this method is that the identity of each user is exposed through the use of open communication channels, thereby providing an opportunity for an adversary to utilize a traceability attack against a valid user. Another area where researchers have provided an alternative is through the development of an authentication mechanism for IoT-based e-health cloud environments by Ayub et al.^5^. An additional feature to this new authentication mechanism was a three-factor authentication model to allow for a secure way to gain access to an entity’s resources. Unfortunately, the authors’ authentication mechanism has a fundamental design flaw in the fact that when a user’s credentials are updated, they are not always consistent with those of the other entities within the same system. Khan et al.^6^ applied blockchain technology along with traditional cryptographic methods to improve the security of e-health services. This improved security comes at a price of increased computational overhead and higher bandwidth usage. Finally, Ansari et al.^7^ presented a privacy-centric framework for cloud-assisted e-healthcare systems. This is a positive advancement in the field, but the application of short-length secret keys and random values creates vulnerabilities to impersonation and DoS attacks making it easier for an attacker to compromise the sensitive credentials of the system.

There was much advocacy for a move away from traditional health care delivery models, to Cloud-enabled health care delivery models Masud et al.^8^, by enabling secure online access to health records through the creation of keys based on mappings to enable encrypted and effective interactions with a cloud-server. Unfortunately, the authors did not formally validate the correctness and robustness of the proposed solution which raises concerns about the reliability of the proposed solution. Padmaja et al.^9^ attempted to address security concerns associated with cloud computing by implementing a protocol that enables patients to access medical services securely and efficiently. Unfortunately, the authors utilized the MD5 hashing function during the device authentication process which has been shown to be susceptible to hash collision attacks and therefore does not provide adequate security assurances for e-healthcare systems. Chandrakar et al.^10^ implemented a cloud-based protocol using a hybrid cryptographic approach to enable remote monitoring of electronic health records and protect patient privacy using mobile devices. Although the authors used a hybrid cryptographic approach and included bilinear pairings and XOR operations, this resulted in significant computational overhead and the protocol remains susceptible to traceability attacks. Deebak et al.^11^ developed an innovative method to authenticate services utilizing bilinear pairings, bio-hashing, XOR operations, and symmetric cryptography techniques. The authors’ model used biometric information to enhance mutual verification for cloud-oriented health platforms. While the authors demonstrated a number of security benefits for the proposed solution, it also has the drawback of having high communication costs because of the computationally intensive modular exponentiation required in pairing-based cryptography.

Chiou et al.^12^, developed an e-healthcare service delivery framework that uses cloud technology to provide both efficient telemedicine services and maintain patient confidentiality, un-linkability, and authenticating messages, via modular exponentiation to reduce computation cost; however, this approach does not resolve critical security issues like key hijacking, server impersonation, or patient anonymity, and it remains at risk for identity guessing attacks that could compromise patient confidentiality. In another area of work, Qadir et al.^13^ proposed a modular system that will allow registered patients to have access to medical documentation from various hospitals. The model, however, has not given sufficient attention to privacy considerations of medical data, resulting in serious ethical concerns for the protection of sensitive patient information. Okikiola et al.^14^ focused on detecting insider threats in cloud-based environments with a model that utilizes watermark extraction and logging mechanisms based on symmetric encryption/decryption. The model effectively identifies fraudulent activities on telemedicine servers, but does not consider the potential risks of unauthorized modification of medical records. Benil et al.^15^ proposed a blockchain enabled model utilizing elliptic curve aggregate certificateless signatures to enable confidentiality and data integrity. Their hybrid model, however, is impractical for use in a real world setting because of the high computation costs associated with the elliptic curve discrete logarithm problem (ECDLP), as well as the need for repetitive exchanges of public keys that requires significant bandwidth.

Alqarni et al.^16^, proposed a lightweight authentication protocol designed for the resource-constrained healthcare environment and demonstrated the success of this protocol in providing secure service to the deployed device. Nevertheless, there is a major weakness in the design of Alqarni et al.’s^16^ protocol; the identity sent from the sensor node to the routing node on the second message sent between them is not encrypted and is therefore in plain view. Therefore, the identities can be accessed by an attacker who launches either an insider attack or a tracking attack and compromises the user’s privacy. Abbasi et al.^17^, have presented a security framework which will allow for efficient provision of access to healthcare services for all participating entities. While Abbasi et al.’s^17^has provided secure two-party authentication, they do not provide any mechanisms to protect patient-level privacy and confidentiality of electronic medical records (EMRs) in the cloud-assisted environment - a fundamental issue they failed to address. In attempting to resolve the security and privacy issues associated with cloud-assisted healthcare systems, Vellaichamy et al.^18^, have highlighted the importance of having quick access to electronic medical records (EMRs) to improve user adoption of cloud-assisted healthcare systems. However, Vellaichamy et al.’s^18^ method fails to provide sufficient protections for patient-identifiable information (PII), such as patient preferences, location and geographic coordinates, which can be compromised using identity spoofing attacks. The authors of^19^, have argued that the use of cloud-based systems will enable efficient and privacy-aware sharing of EMRs. However, the proposed model of^19^ is vulnerable to privacy breaches, because of the lack of adequate protection of user credentials during transmission over public communication channels. Zhang et al.^20^, have proposed a fog-computing-based sharing of health data mechanism, which utilizes edge computing to handle information closer to its source. Zhang et al.’s^20^ method has improved both the privacy of users and the efficiency of the system. However, this method does not accommodate adaptability since the credentials used by Zhang et al.^20^ are generated only once and reused for the duration of the session and thus fail to support dynamic authentication. Finally, Gajmal et al.^21^ have proposed a symmetric encryption based methodology for secure data sharing in healthcare systems.

In 2023, Kumar et al.^22^ proposed RFID based authentication scheme named $ER^2AS$. They employ bitwise XOR, circular left-right rotations and reformation operations for encrypting data. Additionally, Shariq et al.^23^ also made a contribution to cloud security through the introduction of RFID-based authentication technologies. In 2025, Trivedi et al.^24^ proposed an authentication scheme for Internet-of-Health systems. In that they consider three entities as users, the registration gateway and the healthcare server. They also used Shamir’s secret sharing technique, which is the foundation of their scheme. Ghaffar et al.^25^ in the same timeline proposed a machine learning attack resilient authentication protocol for AI-driven remote patient health monitoring by integrating one time physically unclonable function with elliptic curve cryptography. In the same year, Khajehzadeh et al.^26^ found some weaknesses in Masud et al.’s^27^ scheme, such as a replication attack, an insider attack and the absence of a password update phase. To overcome these limitations, the author proposed a new hash function-based authentication approach and provided a password update phase. Additionally, Ghaffar et al.^28^ proposed an access control for an e-healthcare network by utilising hash functions, chaotic maps and physical unclonable functions. During the same period, Saleem et al.^29^ introduced a hash function and physical unclonable function-based scheme for e-healthcare systems. In that they used cipher-block chaining-advanced encryption standard (CBC-AES) for encrypting the important medical data. In the same year, Ghaffar et al.^30^ proposed an ECC-based authentication scheme that uses one-time physical unclonable functions for AI-driven remote patient health monitoring.

Although numerous frameworks, protocols, and schemes have been developed to enhance the privacy, security, and efficiency of electronic health records and data sharing within cloud-assisted e-healthcare systems, many existing cloud-based authentication mechanisms remain susceptible to significant security vulnerabilities. This highlights the pressing need for the development of a robust and secure cloud-based authentication approach specifically designed for healthcare monitoring environments. To address the aforementioned limitations and challenges, this study presents a cloud-powered key agreement system created specifically for safe remote patient monitoring in healthcare systems. The proposed scheme emphasises the preservation of patient privacy and aims to make a meaningful contribution to the e-healthcare domain by enabling the delivery of critical medical services to patients, while simultaneously enhancing the support available to healthcare providers and medical professionals.

**Table 1:** The limitation and authentication techniques of existing work.

**Author and Year**	**Methodology**	**Pros**	**Limitation**
Alzahrani et al.^31^, 2025	ECC and secure hash function-based authentication scheme.	Providing robust authentication and detailed security analysis.	High computation cost.
Deebak et al.^11^, 2020	Developed the bio-hash-based authentication model to ensure mutual trust in cloud-assisted healthcare environments.	Integrated bio-hashing, bilinear pairing and symmetric encryption.	High latency and communication cost.
Hu et al.^32^, 2022	They have used an integrated cryptographic framework and large-scale machine-oriented communication for their protocol.	Mutual authentication and session key agreement.	High computation cost and having privacy issues.
Chandrakar et al.^10^, 2020	Designed a privacy-preserving cloud framework for remote medical monitoring via mobile phones, avoiding in-person hospital visits.	Applied both symmetric and asymmetric cryptographic methods with bilinear mapping and XOR functions.	High computation cost and lacks user anonymity.
Qadir et al.^13^, 2023	Suggested a novel cloud privacy model for e-healthcare using secure authentication and controlled data access.	Employed a Security Secret Key Provider (SSKP) and modular access control approach.	Fails to grant patients direct access to their own medical data.
Lee et al.^33^, 2023	Their scheme uses RFID, physical unclonable function and the secure hash function.	Mutual authentication and RFID technology.	Vulnerable to insider attacks.
Chiou et al.^12^, 2016	Proposed a comprehensive security solution for telemedicine systems, targeting privacy, unlinkability, and data integrity.	Combined bilinear pairing with hashing and XOR operations.	Vulnerable to spoofing and key theft risks.
Benil et al.^15^, 2020	Investigated blockchain-based HER protection using elliptic curves and a novel signature scheme to preserve data confidentiality.	Used ECC for encryption, CAS for signatures, and blockchain to enhance cloud record safety.	High computation cost.
Jan et al.^34^, 2021	Presented a lightweight cryptographic protocol for secure communication in medical IoT using wearable sensors.	Employed a hybrid model combining hashing, XOR operations, and asymmetric encryption.	Vulnerable to traceability issues and insider threats due to identity exposure during key exchange.
Kohli et al.^35^, 2021	They have used DICOM and HL7.FHIR standards for healthcare data interoperability.	Model is effectively managed and easy to access.	Vulnerable to side channel and desynchronization attacks.
Jan et al.^36^, 2023	Offered a simplified authentication mechanism suitable for low-power medical environments and devices.	Relied on basic cryptographic primitives like hashing and XOR gates.	Adequate for minimal systems, but not ideal for multi-party or complex scenarios.
Alzahrani et al.^37^, 2024	Their authentication protocol uses SHA-512 and ECC.	Providing mutual authentication and secure key agreement.	Vulnerable to privileged insider attack.
Padmaja et al.^9^, 2021	The authors addressed security concerns in medical device access within cloud-integrated healthcare, proposing a robust authentication scheme.	Utilised a message digest with hashing and chaotic ordering techniques for device verification.	The scheme misses a concrete and implementable security design for practical deployment.
Nikkhah et al.^38^, 2021	They have used public key cryptography for establishing authentication.	Lightweight authentication	vulnerable to replay and side channel attacks.
Okikiola et al.^14^, 2020	Focused on mitigating insider threats in cloud health systems through traceability and log-based detection mechanisms.	Deployed watermark-based logging and symmetric encryption for integrity monitoring.	The approach does not handle tampering or modifications of records.
Tanveer et al.^39^, 2024	They have used AES and a biometric fuzzy extractor.	Mutual authentication and Scyther simulation.	Vulnerable to key-stolen attack and high computation cost.
Abbasi et al.^17^, 2024	Built a security model for cloud-based healthcare access where users authenticate via a public server.	Implemented ECC alongside biometric fuzzy extraction to protect data.	Provides strong security, but lacks adaptability for multi-entity authentication frameworks.
Masud et al.^8^, 2021	They have used the SHA hash function, the XOR operation and symmetric key encryption/decryption.	Security analysis done by the AVISPA tool.	Vulnerable to tracking attacks.

## Preliminaries

In this section, we provide a brief introduction to the hash function and the cloud server. Furthermore, we discuss the threat model that we have used in the paper.

### Hash function

A cryptographic hash function $h(\cdot )$ is required to satisfy the following security properties:

1. **Preimage resistance:** Given a hash output *H*, it should be computationally infeasible to determine an input *x* such that $h(x) = H$.
2. **Second preimage resistance:** For a given input *x*, it should be hard to find another distinct input $x' \ne x$ for which $h(x) = h(x')$.
3. **Collision resistance:** It must be computationally difficult to find any two distinct inputs $x \ne x'$ that yield the same hash value, i.e., $h(x) = h(x')$.

### Cloud server

The cloud server is at the heart of the E-Healthcare System,and enables integration of wearable devices, sensors, IoT devices, mobile applications, etc. for the collection, processing, and storing of healthcare data and services. The use of the cloud server enables real-time access to medical services by patients and healthcare professionals at an affordable price, which enables immediate tracking and reporting of health conditions. Cloud-based data centers are designed to efficiently process large amounts of healthcare data at high speeds and with reliability; therefore, they enable scalability, privacy, and security for both patients and paramedics and enable timely medical support that can assist in saving lives through life-saving interventions and provide patient transport to hospitals. Additionally, the cloud server facilitates efficient networking, resource management, and emergency medical response systems, which enhance the overall efficiency of e-healthcare systems.

### Threat model

This protocol operates under the Dolev-Yao threat model. In this framework, the adversary is assumed to have complete control over public communication channels. Consequently, they can intercept, modify, or drop transmitted messages, impersonate legitimate participants, and replay previously captured exchanges. Furthermore, if the adversary possesses secret keys—either pre-known or compromised during execution—they can utilise them for encryption or decryption. Nevertheless, any data shared over private channels remains beyond the adversary’s reach, and all entities engaged in the communication are presumed to act honestly.

## Overview of Alzahrani et al.’s protocol

In this phase, we are giving the overview of Alzahrani et al.’s^3^ scheme named “Developing a Provable Secure and Cloud-Centric Authentication Protocol for the e-Healthcare System”. Notations and symbols used for the protocol are shown in Table 2.

**Table 2:** Symbols and notations used in Alzahrani et al.’s^3^ work.

Notation	Description
TTPS	Trusted Third Party Server
PA	Patient
PH	Physician
CS	Cloud Server
p, q	Prime Numbers
$E_P$	Elliptic Curve
$PK_{PH}$	Physician Public Key
$ID_P$	Patient Identity
$ID_{CS}$	Cloud Server Identity
$\oplus$	XOR Operation
$\Delta T$	Matching Function
$\parallel$	Concatenation Function
SK	Session Secret Key
$T_1 \ldots T_{14}$	Different Timestamps
$\lambda$, x, y	Point Over Curve
k	Secret Key
h(.)	Hash Function
$r_1\ldots r_9$	Random Numbers
$n_1\ldots n_9$	Random Nonce
$PK_{PA}$	Patient Public Key
$PK_{CS}$	Cloud Public Key
$ID_{PH}$	Physician Identity

### Setup phase

The trusted third-party server (TTPS) generates unrestricted credentials and a secret key by selecting points over a curve *Eq*(*x*, *y*) with prime order q, using a one-way hash function *h*(.) and secret key $k \in Z_q^*$. The final parameters produced are ${Eq(x, y), \lambda , k, h(.)}$.

### Registration phase

In this phase, the patient, the physician and the cloud server register themself in the trusted third-party server (TTPS).

#### Patient registration phase

**Step 1 -** The patient selects an identity (IDP) and a random number $n_1 \in Z_q^*$, computes $HIDP = h(IDP||n_1)$, and securely sends $\{HIDP, IDP\}$ to the trusted third-party server (TTPS) via a private channel.

**Step 2 -** The trusted third-party server (TTPS) selects a random number $r_{m1} \in Z^*_q$, calculates $PKP = r_{m1} \cdot \lambda$, $HP = h(IDP||PKP)$, and $SP = (r_{m1} \oplus HP) || k$. It then transmits *PKP*, *SP*, *HP*, *k* to the patient securely.

**Step 3 -** Upon receiving the message $\{PKP, SP, HP, k\}$, the patient stores the parameters $\{PKP, SP, HP, k\}$ in its memory, as demonstrated in registration phase 1.

#### Physician registration phase

The following sequence of steps is used to complete this phase:

**Step 1 -** To begin, the physician selects an identity $ID_{PH}$ and picks a random number $n_2 \in \mathbb {Z}_q^*$. Then, the physician computes $HID_{PH} = h(ID_{PH} \parallel n_2)$, and sends the pair $\{HID_{PH}, ID_{PH}\}$ to the trusted third party server (TTPS) through a secure communication channel.

**Step 2 -** The trusted third party server (TTPS) generates a random value $r_{m2} \in \mathbb {Z}_q^*$, computes the physician’s public key as $PK_{PH} = r_{m2} \cdot \lambda$, then derives $H_{PH} = h(ID_{PH} \parallel PK_{PH})$. It further constructs $S_{PH} = (r_{m2} \oplus H_{PH}) \parallel k$, and transmits the message $\{PK_{PH}, S_{PH}, H_{PH}, k\}$ to the physician using a secure channel.

**Step 3 -** Upon receiving the credentials $\{PK_{PH}, S_{PH}, H_{PH}, k\}$, the physician stores them in local memory, completing step 2 of the registration phase.

#### Cloud server registration phase

The steps outlined below are followed to complete this phase:

**Step 1 -** The cloud server begins by choosing an identity $ID_{CS}$ and a random number $n_3 \in \mathbb {Z}_q^*$. It then computes $HID_{CS} = h(ID_{CS} \parallel n_3)$ and securely sends the pair $\{HID_{CS}, ID_{CS}\}$ to the trusted third-party server (TTPS) via a private communication channel.

**Step 2 -** The trusted third-party server (TTPS) generates a random value $r_{m3} \in \mathbb {Z}_q^*$, then computes the cloud server’s public key as $PK_{CS} = r_{m3} \cdot \lambda$. Next, it derives $H_{CS} = h(ID_{CS} \parallel PK_{CS})$ and forms $S_{CS} = (r_{m3} \oplus H_{CS}) \parallel k$. Finally, it transmits the set $\{PK_{CS}, S_{CS}, H_{CS}, k\}$ back to the cloud server using a secure channel.

**Step 3 -**After receiving the message $\{PK_{CS}, S_{CS}, H_{CS}, k\}$, the cloud server stores these credentials in its memory, thereby completing the third step of the registration phase.

### Authentication phase

This phase is carried out through the following three distinct sub-phases:

#### Physician (PH) and Patient (PA)

The completion of this phase involves the following sequence of steps:

**Step 1 -** To initiate the process, the physician picks a random value $n_4 \in \mathbb {Z}_q^*$ and notes the timestamp $T_1$. The physician then calculates $X_{PH} = (n_4 \cdot \lambda ) \parallel T_1$, and transmits the set $\{PK_{PH}, HID_{PC}, X_{PH}, T_1\}$ to the patient via a public communication channel.

**Step 2 -** Upon receiving the message $$\{PK_{PH}, HID_{PC},$$$$X_{PH}, T_1\}$$, the patient verifies the freshness of the timestamp by checking if $$T_2-T_1 \le \Delta T$$. The patient then selects a random number $$r_{m4} \in \mathbb {Z}_q^*$$ and computes $$X_{PH1} =$$$$(r_{m4} \cdot \lambda ) \parallel T_1$$. Next, the patient calculates $$PK_{PH1} =$$$$R_{PH} \oplus h(HID_{PC} \parallel R_{PH} \parallel PK_{PH})$$, and constructs $$PK_S1 =$$$$SP \parallel (X_{PH} \oplus r_{m4}) \parallel PK_{PH1}$$. Then, the patient derives $$PK_T1 = r_{m4} \cdot X_{PH}$$, $$SK =$$$$h(PK_S \parallel PK_T)$$, and $$PK_U1 =$$$$h(SK \parallel X_{PH})$$. Finally, the patient sends the message $$\{HID_P, PK_{PH1}, X_{PH1}, PK_{U1}, T_3\}$$ back to the physician through a public channel.

**Step 3 -** The physician begins by verifying the timestamp to ensure that $T_4 - T_3 \le \Delta T$. Then, the physician computes $PK_P = RP \oplus h(HID_P \parallel RP \parallel PK_P)$. Using this, the physician forms $PK_{V1} = SPH \parallel (XP \oplus n_4) \parallel PK_P$, and calculates $PK_{W1} = n_4 \cdot XP$. Next, the session key is derived as $SK = h(PK_{V1} \parallel PK_{W1})$, and a verification tag is computed as $PK^*_{U1} = h(SK \parallel X_{PH})$. The physician compares $PK^*_{U1}$ with the received $PK_{U1}$; if they match, the physician accepts *SK* as the shared session key, completing the mutual authentication phase shown in phase/Fig. 4.

#### Physician (PH) and Cloud Server (CS)

This sub-phase involves the following computational steps to enable mutual authentication between the parties:

**Step 1 -** To start, the physician chooses a random number $n_5 \in \mathbb {Z}_q^*$, notes the timestamp $T_5$, and computes $X_{PH1} = (n_5 \cdot \lambda ) \parallel T_5$. The physician then sends the message $\{PK_{PH}, HID_{PH}, X_{PH}, T_5\}$ to the cloud server through an unsecured communication channel.

**Step 2 -** Once the cloud server receives the message $$\{PK_{PH}, HID_{PH}, X_{PH}, T_5\}$$, it checks the validity of the timestamp by ensuring $$T_6 - T_5 \le \Delta T$$. It then selects a random number $$r_{m5} \in \mathbb {Z}_q^*$$ and computes $$X_{PH2} =$$$$(r_{m5} \cdot \lambda ) \parallel T_5$$. Following that, the cloud server calculates $$PK_{HP2} =$$$$R_{PH} \oplus h(HID_{PC} \parallel R_{PH} \parallel PK_{PH})$$, constructs $$PK_{S2} = S_{CS} \parallel (X_{PH2} \oplus r_{m5}) \parallel PK_{HP2}$$, and derives $$PK_{T2} = r_{m5} \cdot X_{PH2}$$. The session key is then computed as $$SK = h(PK_{S2} \parallel PK_{T2})$$, and the authentication tag as $$PK_{U2} = h(SK \parallel X_{CS})$$. Finally, the server sends the message $$\{HID_{CS}, PK_{PH2}, X_{PH2}, PK_{U2}, T_7\}$$ to the physician over an open channel.

**Step 3 -** The physician begins by validating the timestamp, ensuring that $T_8 - T_7 \le \Delta T$. Then, the physician computes $PK_{CS} = R_{PH} \oplus h(HID_{CS} \parallel R_{CS} \parallel PK_{CS})$, and constructs $PK_{V2} = S_{CS} \parallel (X_{PH} \oplus n_5) \parallel PK_{PH}$. Next, the physician calculates $PK_{W2} = n_5 \cdot X_{CS}$ and derives the session key as $SK = h(PK_{V2} \parallel PK_{W2})$. A verification value is then generated as $PK^*_{U2} = h(SK \parallel X_{CS})$, which is compared against the received $PK_{U2}$. If the values match, the physician accepts *SK* as the shared session key, thus completing the mutual authentication phase (Fig. 5).

#### Cloud Server (CS) with Patient (PA)

This sub-phase involves the following computational steps to establish mutual authentication between the entities:

**Step 1 -** The cloud server chooses a random number $n_6 \in \mathbb {Z}_q^*$, computes $X_{CS2} = (n_6 \cdot \lambda ) \parallel T_9$, and transmits the message $\{HID_{CS}, R_{CS}, X_{CS1}, T_9\}$ to the patient via an open communication channel.

**Step 2 -** The patient begins by verifying the timestamp to confirm that $T_{10} - T_9 \le \Delta T$. Then, the patient selects a random number $r_{m6} \in \mathbb {Z}_q^*$ and calculates $X_{CS2} = r_{m6} \cdot X_{CS1}$. Next, the patient computes $PK_{CS2} = R_{CS} \oplus h(HID_{PC} \parallel R_{PH} \parallel PK_{PH})$, and forms $PK_{S3} = (SP \parallel (X_{CS2} \oplus r_{m6})) \parallel PK_{CS2}$. The patient also calculates $PK_{T3} = r_{m6} \cdot X_{CS2}$, derives the session key as $SK = h(PK_{S3} \parallel PK_{T3})$, and computes the authentication tag $PK_{U3} = h(SK \parallel X_{CS2})$. Finally, the patient sends the message $\{HID_P, PK_{CS2}, X_{CS2}, PK_{U3}, T_{11}\}$ to the cloud server via an open channel.

**Step 3 -** The cloud server verifies the timestamp by checking that $T_{12} - T_{11} \le \Delta T$. It then computes $PK_{CS2} = R_{CS} \oplus h(HID_{CS} \parallel R_{CS} \parallel PK_{CS})$, constructs $PK_{V3} = S_{CS2} \parallel (X_{CS2} \oplus n_6) \parallel PK_{CS2}$, and calculates $PK_{W3} = n_6 \cdot X_{CS2}$. Next, the session key is derived as $SK = h(PK_{V3} \parallel PK_{W3})$, and a verification tag $PK^*_{U3} = h(SK \parallel X_{CS})$ is generated. The server compares $PK^*_{U3}$ with the received $PK_{U3}$, and upon a successful match, stores *SK* as the session secret key, shown in the mutual authentication Fig. 6.

### Cryptanalysis of Alzahrani et al.’s protocol

In this subsection, we will discuss about the shortcomings of Alzahrani et al.’s^3^ protocol.

#### Physician impersonation attack in PH-PA authentication phase

In this phase, the patient does not perform any verification of the physician. As a result, an attacker can impersonate the physician, thereby enabling a potential impersonation attack. The step-by-step cryptanalysis is presented as follows:

**Step 1 -** Since the patient does not verify $X_{\text {PHA}}$, an attacker can generate a forged value $X_{\text {PHA}} = (r_A \cdot \lambda ) \parallel n_A$, where $r_A \cdot \lambda$ is an elliptic curve point chosen by the attacker and $n_A$ is a randomly selected nonce. The attacker then sends the tuple $\{PK_{\text {PHA}}, HID_{\text {PCA}}, X_{\text {PHA}}, T_{1A}\}$ to the patient, where $PK_{\text {PHA}}$ is the attacker’s counterfeit public key.

**Step 2 -** After receiving the physician’s parameters, the patient only verifies the timestamp condition $(T_2 - T_1 \le \Delta T)$ and then blindly proceeds to compute $PK_{T1} = r_{m4} \cdot X_{\text {PHA}}$ and $SK = h(PK_{S1} \parallel PK_{T1})$. Since $X_{\text {PHA}}$ is generated by the attacker, the attacker can similarly compute $PK'_{T1} = r_A \cdot X_{\text {PHA}}$ and derive the session key as $SK' = h(PK'_{S1} \parallel PK'_{T1})$. Consequently, the attacker knows the session key and can decrypt the patient’s confidential data, which can then be exploited for malicious purposes.

#### Cloud server impersonation attack in CS-PA authentication phase

In the CS-PA phase, the cloud server sends the tuple $\{HID_{\text {CS}}, PK_{\text {CS2}}, X_{\text {CS1}}, T_9\}$ to the patient. Upon receiving these parameters, the patient directly computes the session key without verifying $HID_{\text {CS}}$. As a result, an attacker can easily impersonate the cloud server. The following steps demonstrate how such an impersonation can be carried out.

**Step 1 -** The attacker selects a fake nonce $n_A \in \mathbb {Z}_q^*$ and computes $X_A = (n_A \cdot \lambda ) \parallel T_A$. The attacker then sends the tuple $\langle HID_A, PK_A, X_A, T_A \rangle$ to the patient.

**Step 2 -** After receiving the cloud server’s parameters, the patient does not verify $HID_{\text {CS}}$ and blindly computes $PK_{T3} = r_{m6} \cdot X_A$, followed by the session key using the attacker’s values as $SK_A = h(PK_{S3} \parallel PK_{T3})$. Since $SK_A$ is computed using the attacker’s parameters, the attacker can decrypt the patient’s data and intercept or modify the messages exchanged between the patient and the cloud server.

#### Physician impersonation attack in PH-CS authentication phase

In this phase, the physician sends the tuple $\langle PK_{\text {PH1}}, HID_{\text {PH}}, X_{\text {PH1}}, T_5 \rangle$ to the cloud server over a public channel. Upon receiving these parameters, the cloud server does not verify $PK_{\text {PH1}}$ before responding. It only checks the timestamp condition $(T_6 - T_5 \le T)$ and then selects a random number $r_{m5}$ to compute $X_{\text {CS1}} = (r_{m5} \cdot \lambda ) \parallel T_5$. The following steps demonstrate how such an impersonation can be carried out.

**Step 1 -** An attacker can select a fake nonce $n_A \in \mathbb {Z}_q^*$ and compute $X_A = (n_A \cdot \lambda ) \parallel T_A$. The attacker then sends the forged tuple $\langle PK_A, HID_A, X_A, T_A \rangle$ to the cloud server.

**Step 2 -** After receiving the physician’s parameters, the cloud server blindly accepts $PK_A$ and computes the session key based on the attacker’s parameters as $SK_A = h(PK_{VA} \parallel PK_{WA})$. As a result, the attacker can authenticate as a legitimate physician to the cloud server. Consequently, the attacker gains full access to the cloud server and can retrieve or manipulate the patient’s sensitive medical records.

## Proposed work

The new method for authenticating a user and agreeing on keys to be used for that session provides an additional layer of security beyond authentication by providing a way to ensure access control and provide privacy. The proposed scheme also includes mechanisms to handle the process of transferring ownership of the user ID, updating passwords and updating biometric information of users.

The proposed protocol includes seven different phases: the initial setup phase with the overall system model, the registration phase and the login phase, the authentication phase, the ownership transfer phase, the password update phase, and the biometrics update phase. Symbols and notations used in the proposed work are presented in Table 3.

### Setup phase and system model

**Fig. 1:**
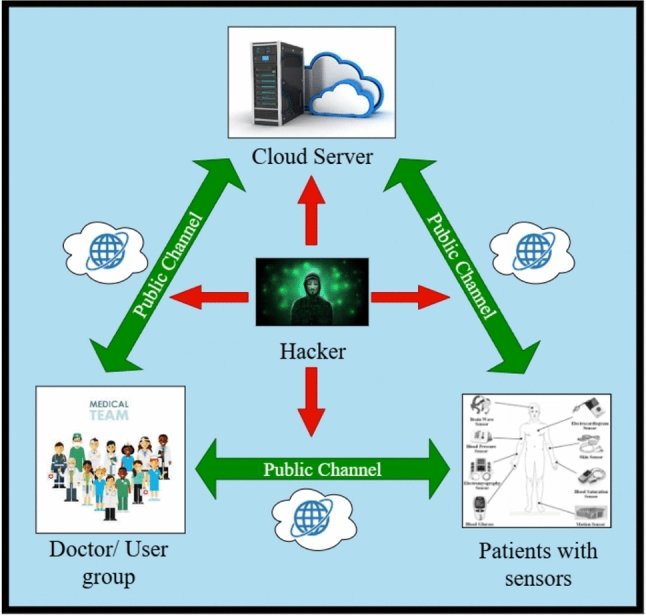
Cloud-based e-healthcare architecture.

Paramedical workers (doctors, nurses, etc.) and patients have to enroll into the cloud enabled service to be able to use the e-Healthcare Service. At that time, credentials of the patient’s side IoT devices (sensors, wearables), as well as the mobile devices of paramedical workers, will be saved securely at the Cloud Server. The Cloud Server is the central element in the system, providing a safe space for managing and processing all confidential data about patients and paramedical workers. Once patients have registered, they can establish contact with physicians using the Cloud Server to receive diagnostic opinions and treatment advice. Thus, the amount of information is reduced to a minimum. It enables rapid assessment of patient health status by physicians and provides effective treatment. The proposed cloud assisted key agreement framework architecture, shown in Fig. 1 and described in this section, has three main elements: the patient, who is equipped with internal sensors or wearable devices to collect real time physiological data; the cloud server, responsible for the deployment, networking and secure saving of the data; the paramedical staff, represented by physicians for direct medical evaluation and treatment or nurses for patient care. Real-time physiological data is continuously sent to the cloud-assisted e-Healthcare System. Physicians can access it easily from their devices via the cloud data center.

**Assumption -** To carry out the security analysis for the new protocol and to develop its architecture, we use the following assumptions-

- We assume that the memory of the sensors will be tamper-resistant (i.e., an attacker cannot get the long-term secrets on a sensor from it through a physical attack).
- We assume that the cloud server will keep the data securely; specifically, the integrity and confidentiality of the data being kept at the cloud will not be compromised by either server-side breaches or malicious insiders.

#### Doctor node

Mobile devices and smart cards integrated in the doctor or user node of e-Healthcare systems represent a major improvement that will significantly enhance the ability of health care providers to deliver quality services. The integration of these devices in e-healthcare systems enables providers to offer faster service through easier data access; real time communication with other users and the ability to monitor patients remotely. Mobile devices are used extensively, with high functionality; therefore, they provide an excellent opportunity for healthcare professionals to improve patient outcomes. Moreover, this approach ensures secure access to medical data, supports continuous monitoring, and enhances communication between patients and physicians, thereby improving both patient care and overall operational efficiency.

#### Patient/sensor

Biological sensors (which combine biological with technological components) provide the opportunity for the exact and portable on-site determination of many analytes in “real time”. Biological sensors have dramatically improved medical diagnostic capabilities, patient monitoring, and disease management, and therefore represent an important resource in contemporary medicine. The continuous development of nanotechnology, novel materials and data analysis also enhances their importance within various disciplines. As physicochemical detectors coupled with biological detectors, biological sensors may be used to determine whether a substance is present and/or its concentration, often in real time. The use of biological sensors extends into areas outside of the medical field including biotechnology, food quality assurance, and environmental monitoring. In a medical setting, biological sensors are essential to the tracking of physiological parameters, diagnosis of diseases and management of chronic health issues.

Embedded sensors within the human body allow uninterrupted monitoring by gathering and transmitting essential physiological data to cloud servers, making them a cornerstone of healthcare technology. Examples include temperature sensors for body heat regulation, pressure sensors to measure breathing time or identify stress on the jaw and central nervous system, oxygen saturation sensors to track blood oxygen levels, and optical sensors to evaluate vision. Moreover, advanced tools like MRI, EEG, and ECG sensors evaluate cardiac plus neurological processes, whereas ventilator sensors guarantee constant oxygen delivery. Collectively, these biosensors and devices generate a steady flow of medical records that guarantees consistent and comprehensive care for patients.

**Table 3:** Symbols and notations used in the proposed work.

Notation	Description
*CS*	Cloud server
$D_{m}$	$m_{th}$ doctor/user
$ID_{m}$	Identity of the $m_{th}$ doctor/user
$PID_n$	Identity of the $n_{th}$ patient/sensor
*CMK*	Master key of the cloud server
$KID_m$	Session key of the doctor node
$CSK_n$	Session key of the medical server
$IDPK_n$	Session key of the sensor node
$SKS_n$	Secret key of the sensor node
$r_{m1}, r_{m4}$	Random numbers generated by the doctor
$r_{m2}, r_{m5}$	Random numbers generated by the server
$r_{m3}, r_{m6}$	Random numbers generated by the sensor
$T_1, T_2, T_3, T_4, T_5$	Current time stamps
$\wedge$	AND operation
$\oplus$	XOR operation
||	Concatenation operation

### Doctor registration phase

**Fig. 2:**
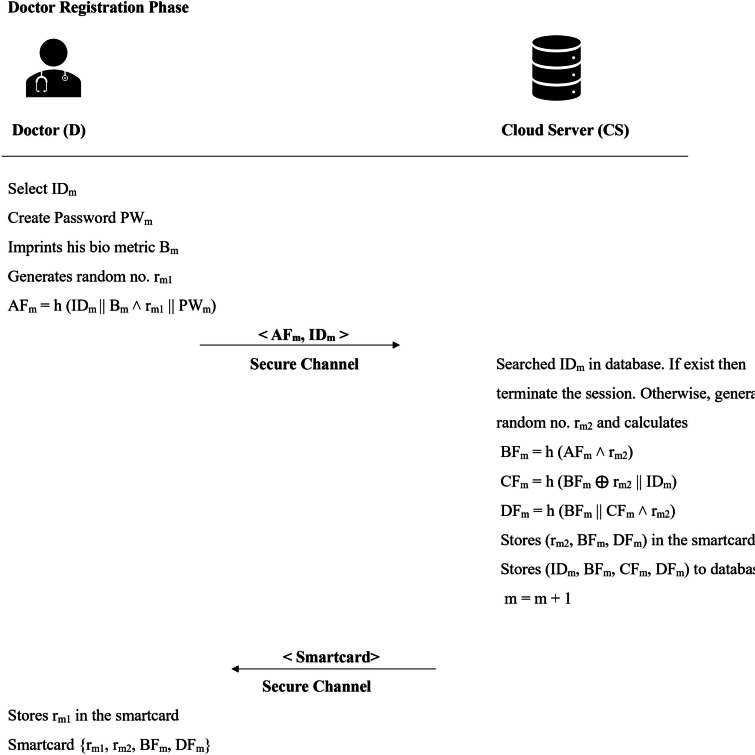
Doctor registration phase.

When a new doctor $D_m$ becomes part of the system, he/she register with the cloud server and receive a smart card for future logins. The system maintains a count of registered doctors, which starts at zero and increments by one with each new registration. This procedure is illustrated in Fig. 2 and elaborated upon in the following explanation.

**Step 1 -** Doctor $D_m$ begins by selecting a user identity $ID_m$, imprints his/her biometrics $B_m$, a password $PW_m$, and a random value $r_{m_1}$. Using these, the doctor computes $AF_m = h(ID_m || B_m \wedge r_{m_1} || ID_s)$. The doctor then securely sends $\langle AF_m, ID_m \rangle$ to the cloud server for registration.

**Step 2 -** After receiving the registration request, the server first checks its database to determine whether the chosen identity $ID_m$ has already been registered. If a match is found, the server terminates the session. If no existing entry is found, the server proceeds by generating a random number $r_{m_2}$ and performing the following computations:

- $$BF_m = h(AF_m \wedge r_{m_2}).$$
- $$CF_m = h(BF_m \oplus r_{m_2}||ID_m ).$$
- $$DF_m = h(BF_m||CF_m \wedge r_{m_2}).$$

The server then stores the values $\langle r_{m_2}, BF_m, DF_m \rangle$ on a smartcard, which is issued to the doctor. Additionally, it records $\langle ID_m, BF_m, CF_m, DF_m \rangle$ in its secure, tamper-proof database. Finally, it updates the registration counter by one to indicate that a new doctor has successfully signed up. The smartcard is securely handed over to the doctor.

**Step 3 -** The smartcard integrates the initially selected random number $r_{m_1}$ into its memory. Once this step is completed, it securely stores the set $\langle r_{m_1}, r_{m_2}, BF_m, DF_m \rangle$.

### Patient/sensor registration phase

**Fig. 3:**
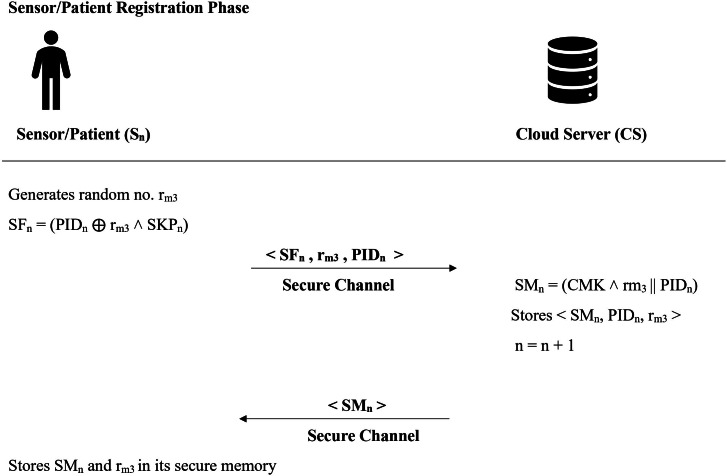
Sensor registration phase.

Patients, referred to as sensors ($S_n$), have the option to register with the cloud server. The system uses the variable *n* to monitor the total number of patients who have signed up. Initially, $n = 0$, and it increments by one with each successful registration. Figure 3 presents a visual overview of this process, which is explained in detail below.

**Step 1 -** At the start, sensor $S_n$ selects a random number $r_{m_3}$ and then calculates $SF_n = h(PID_n \oplus r_{m_3} \wedge SKP_n)$, where $PID_n$ is the unique identifier of the sensor (patient), and $SKP_n$ is the sensor’s secret key. The sensor then securely sends the triplet $\langle PID_n, r_{m_3}, SF_n \rangle$ to the cloud server.

**Step 2 -** Upon receiving the registration request, the server carries out the following steps:

- It computes $SM_n = h (CMK \wedge r_{m_3} || PID_n)$, where $PID_n$ denotes the sensor’s (patient’s) unique ID, and *CMK* is the server’s master secret key.
- The server securely stores the tuple $\langle SF_n, PID_n, r_{m_3} \rangle$ in its tamper-resistant database.
- It then increments the variable *n* by one to account for the new patient registration. Finally, the computed value $SM_n$ is securely sent back to the sensor.

**Step 3 -** The sensor safely stores the pair $\langle SM_n, r_{m_3} \rangle$ in its tamper-resistant memory for future use.

### Login phase

Once the smartcard is inserted, the doctor $D_m$ inputs his/her credentials: user ID $ID^{*}_{m}$, biometrics ${B^*_m}$ and password $PW^{*}_{m}$. The smartcard then performs a series of computations using these credentials and previously stored values. First, it calculates $AF^*_m = h(ID^*_m\parallel B^*_m \wedge r_{m_1}\parallel PW^*$), where $r_{m_1}$ is the initial random number chosen by the doctor. Next, the smartcard computes $BF^{*}_{m} = h(AF^*_m \wedge r_{m_2})$, where $r_{m_2}$ is the server’s random value issued during registration. Using the value $BF^{*}_{m}$, it then calculates $CF^{*}_{m} = h(BF^*_m \oplus r_{m_2}\parallel ID^*_m)$ and finally $DF^{*}_{m} = h(CF^{*}_{m}\parallel BF^*_m \wedge r_{m_2}).$ The smartcard checks whether this computed $DF^{*}_{m}$ matches the stored value $DF_{m}$. If they match, it confirms the legitimacy of the user and the integrity of the smartcard.

Upon successful verification, the smartcard generates a new random number $$r_{m_4}$$ and records the current timestamp $$T_1$$. It then computes: $$SD_{1m} =$$$$h(DF_{m}\parallel CF_{m} \parallel T_1) \oplus r_{m_4}$$, $$SD_{2m} =$$$$h(DF_{m} \wedge (SD_{1m} \parallel r_{m_4}) \oplus PID_n$$, where $$PID_n$$ is the patient’s identifier, finally, the smartcard sends the set $$\langle T_{1}, SD_{2m}, SD_{1m}\rangle$$ to the cloud server as part of the login request.

### Authentication phase

**Step 1 -** Upon receiving the login request, the server first records the current timestamp $$T_2$$ and verifies the freshness of the request by checking whether $$| T_2-T_1 | < \Delta T$$. If this condition is satisfied, the server proceeds with the following computations. It first calculates $$r_{m_4}^{*} = h(DF_{m} \wedge CF_{m} \parallel T_1) \oplus SD_{1m} .$$ Then, using the derived $$r_{m_4}^{*}$$, it computes $$PID_n^{*} = h(DF_{m} \wedge (SD_{1m} \parallel r_{m_4}^{*}) \oplus SD_{2m}$$, and finally derives $$SD_{2m}^{*} = h(DF_{m} \wedge SD_{1m} \parallel r_{m_4}^{*}) \oplus PID_n^{*}.$$ The server checks whether the calculated $$SD_{2m}^{*}$$ is equal to the received $$SD_{2m}$$. If this equality holds, it confirms the user’s authenticity. Following successful authentication, the server generates a new random number $$r_{m_5}$$ and computes: $$SE_n =$$$$h(PID_n \wedge r_{m_3} \parallel SF_n) \oplus r_{m_5}$$, $$SM_n =$$$$h(CMK \wedge r_{m_3} \parallel PID_n)$$ and $$SN_n =$$$$h(SM_n \wedge SF_n \parallel r_{m_5}) .$$ Finally, the server sends the tuple $$\langle T_2, SE_n, SN_n \rangle$$ to the patient (sensor).

**Step 2 -** To verify the freshness of the received message from the server, the sensor generates a new timestamp $T_3$ and checks whether the condition $|T_3 - T_2| < \Delta T$ is satisfied. If the condition holds, the sensor proceeds with the following computations. First, it computes $r_{m_5}^{*} = h(PID_n \wedge r_{m_3} \parallel SF_n) \oplus SE_n.$ This value is derived to retrieve the random number $r_{m5}$ used by the server. Then, the sensor generates a new random number $r_{m_6}$ and calculates $SF_n^{*} = h(PID_n \oplus r_{m_3} \wedge SKP_m)$ and $SN_n^{*} = h(SM_n \wedge SF_n^{*} \parallel r_{m_5}^{*}).$ The sensor then checks whether the computed $SN_n^{*}$ matches the received value $SN_n$. If they are equal, the server is authenticated. After verifying the server’s authenticity, the sensor proceeds to calculate $IDPK_n = h(PID_n \wedge r_{m_5} \parallel r_{m_6}).$ It then computes $SX_n = h(SF_n \wedge SM_n \parallel r_{m_3}) \oplus r_{m_6}$ and $SD_n = h(T_3 \wedge IDPK_n \parallel SF_n).$ Finally, the sensor sends the message $\langle T_3, SX_n, SD_n \rangle$ back to the server to complete its part of the mutual authentication process.

**Step 3 -** To confirm the freshness of the received message and authenticate the sensor, the server first generates a new timestamp $T_4$ and checks whether the condition $| T_4-T_3 | < \Delta T$ is satisfied. If the condition holds, the server continues with the authentication process by computing the following:

$r_{m_6}^{*} = h(SF_n \wedge SM_n \parallel r_{m_3}) \oplus SX_n.$ This value helps in reconstructing the random number $r_{m_6}$ used by the sensor. Then the server computes the session key: $CSK_n^{*} = h(PID_n \wedge r_{m_5} \parallel r_{m_6}^{*})$ and also computes: $SD_n^{*} = h(T_3 \wedge IDPK_n \parallel SF_n).$ The server then checks whether the computed value $SD_n^{*}$ matches the received value $SD_n$. If the values are equal, the message is authenticated and confirmed to be from the legitimate sensor. Next, the server proceeds with mutual authentication towards the user. It calculates: $So_{m} = (AF_m \oplus r_{m_5})$, $Sn_{m} = (AF_m \oplus r_{m_6})$ and $SD_{3m} = h(AF_{m} \wedge So_{m} \parallel Sn_{m} \parallel T_4).$

Finally, the server sends the set $\langle SD_{3m}, T_{4}, So_{m}, Sn_{m} \rangle$ to the user to complete the authentication cycle.

**Step 4 -** Upon receiving the message from the server, the user ensures the message’s timeliness by generating a new timestamp $T_5$ and verifying that the condition $|T_5 - T_4| < \Delta T$ holds. If the condition is satisfied, the user proceeds with computations to authenticate the server. First, the user reconstructs the values used by the server: $r_{m_5}^{*} = AF_{m} \oplus So_{m}$, $r_{m_6}^{*} = AF_{m} \oplus Sn_{m} .$ Then, the user recalculates: $SD_{3m}^{*} = h(AF_{m} \wedge So_{m} \parallel Sn_{m} \parallel T_4).$ Then the user compares $SD_{3m}^{*}$ with the received $SD_{3m}$. If they are identical, the server is considered authenticated.

**Fig. 4:**
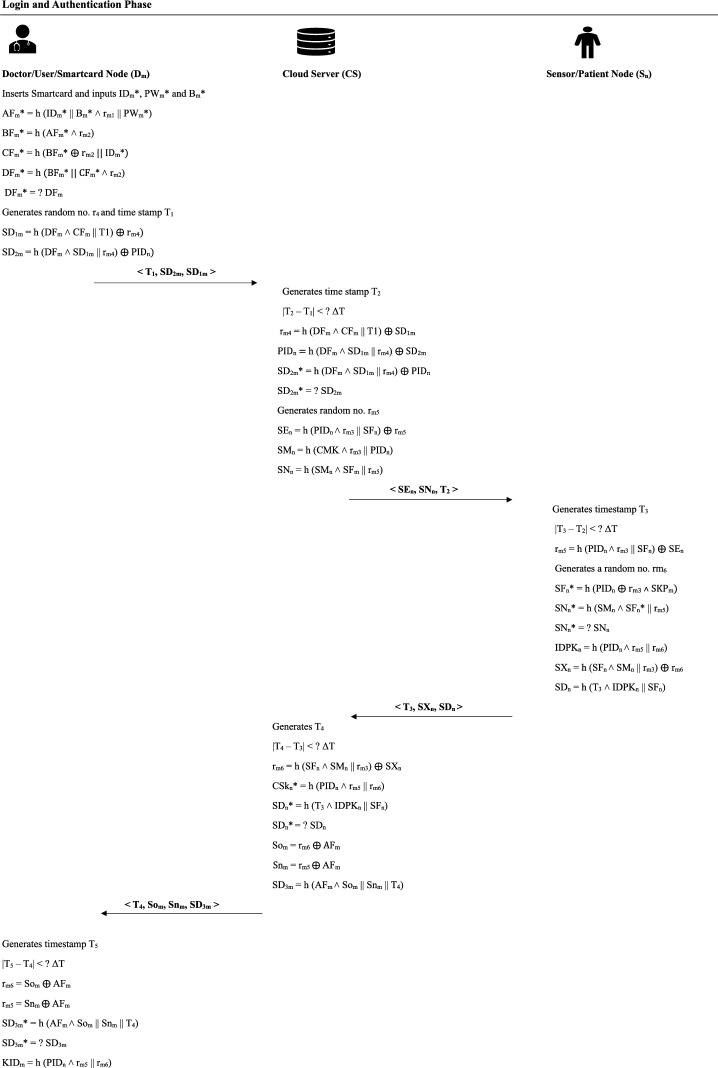
Login and authentication phase.

Finally, the user derives the session key as: $KID_m = h(PID_n \wedge r_{m_5} \parallel r_{m_6}) .$ This key $KID_m$ is used as the session secret for further secure communication. Figure 4 presents a visual overview of the login and authentication process.

### Ownership transfer phase

**Fig. 5:**
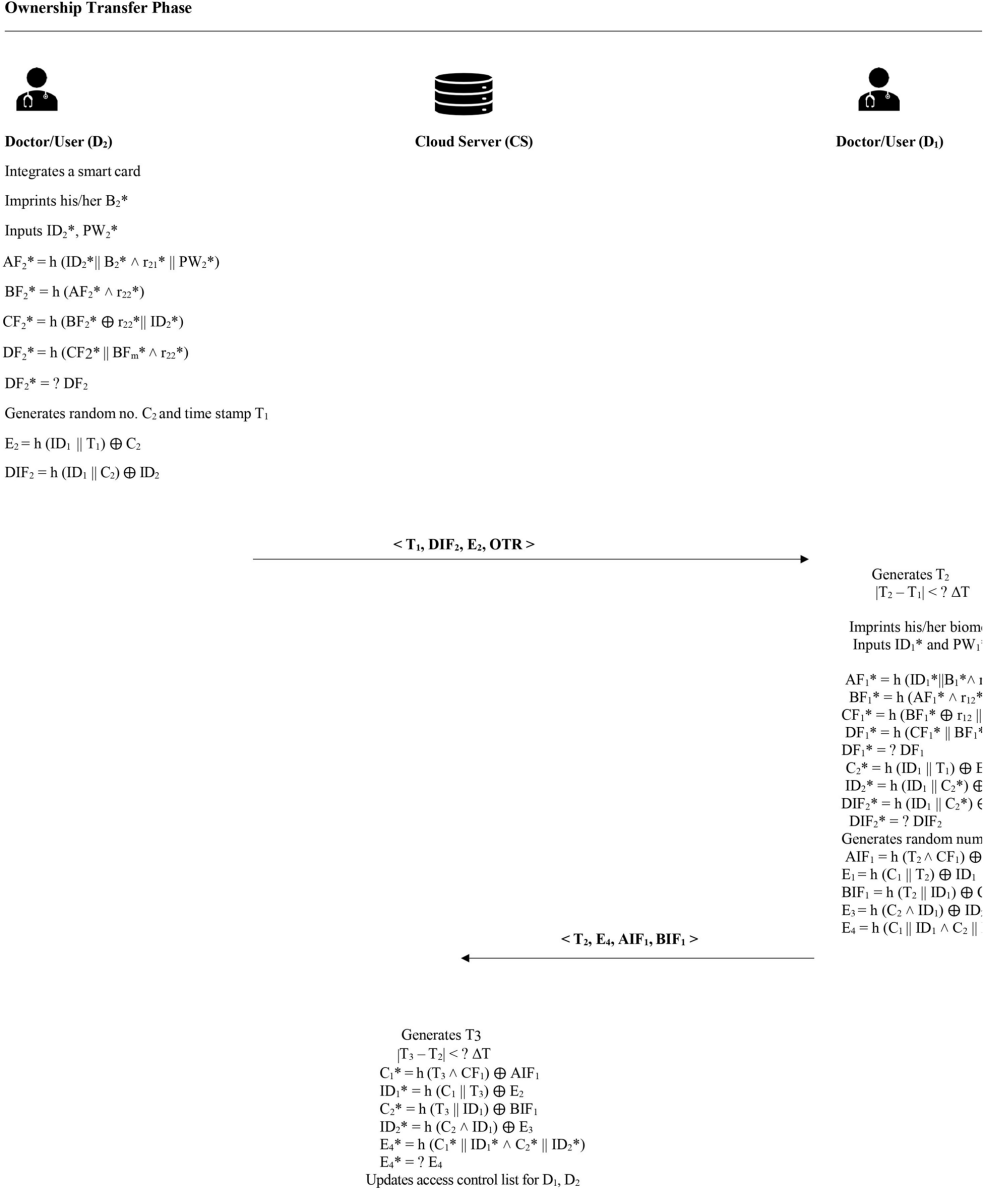
Ownership transfer phase.

In this phase, a transfer of access rights takes place between two physicians concerning a specific patient’s data monitored through the $n^{th}$ sensor. Initially, Doctor $D_1$ holds the authority to access this patient’s medical information. However, through a secure interaction, $D_1$ delegates this privilege to another physician, Doctor $D_2$. Once the process concludes, $D_1$ no longer retains access to the data from the $n^{th}$ sensor, and $D_2$ becomes the new authorised user. This transition of access rights is securely performed and is visually represented in Fig. 5.

**Step 1 -** To initiate the transfer process, the second doctor, $D_2$, begins by inserting his smart card and entering his login credentials—namely, his identity ${ID}_2^*$, biometrics $B^*_2$ and password ${PW}_2^*$. The smart card then verifies $D_2$’s legitimacy by computing a set of values: $AF_2^*= h(ID^*_2 \parallel B^*_2 \wedge r_{2_1} \parallel PW^*_2)$, $BF_2^*= h(AF^*_2 \wedge r_{2_2})$, $CF_2^*= h(BF^*_2 \oplus r_{2_2} \parallel ID^*_2)$ and $DF_2^*= h(CF_2^*\parallel BF^*_2 \wedge r_{2_2}).$

It then checks whether the computed $DF_2^*$ matches the stored value $DF_2$. If there is a mismatch, access is denied, and the card is rejected. However, if the values match, the smart card proceeds by generating a fresh random number $C_2$ and a timestamp $T_1$. Next, it computes: $E_1 = h({ID}_1 \parallel T_1)\oplus C_2$ and ${DIF}_1 = h({ID}_1 \parallel C_2) \oplus {ID}_2$, where ${ID}_1$ is the identifier of the first doctor $(D_1)$. With these values, $(D_2)$ prepares and sends an ownership transfer request message in the form of $\langle T_1, DIF_1, \text {OTrequest} \rangle$ to $(D_1)$ via the cloud server.

**Step 2 -** Upon receiving the ownership transfer message, the first doctor $(D_1)$ begins by checking the freshness of the received data. To do this, he generates a new timestamp $T_2$ and verifies the condition $|T_2 - T_1| < \Delta T$. If this condition holds, $(D_1)$ proceeds by inserting his smart card and entering his credentials: ${ID}_1^*$, $B^*_1$ and ${PW}_1^*$. To authenticate himself and confirm that the smart card belongs to $(D_1)$, the smart card performs the following computations: $AF_1^*= h(ID^*_1 \parallel B^*_1 \wedge r_{1_1} \parallel PW^*_1)$, $BF_1^*= h(AF^*_1 \wedge r_{1_2})$, $CF_1^*= h(BF^*_1 \oplus r_{1_2} \parallel ID^*_1)$ and $DF_1^*= h(CF_1^*|| BF^*_1 \wedge r_{1_2}).$

The smart card then checks whether the computed value $D_1^*$ matches the stored value $D_1$. A match confirms the legitimacy of D1 as the card owner. Next, to authenticate $D_2$ as the sender of the original ownership transfer request, the smart card computes: $C^*_2 = h(ID_1 \parallel T_1)\oplus E_1$, $ID_2^*= h(ID_1 \parallel C^*_2) \oplus DIF_1$ and $DIF_1 = h(ID_1 \parallel C^*_2)\oplus ID^*_2$. $D_1$ then verifies if the $DIF_2$ received matches the calculated value of $DIF^*_2$, confirming that the message originated from $D_2$. Following this verification, $D_1$ selects a new random number $C_1$ and computes the following: ${AIF}_1 = h(T_2 \wedge CF_1) \oplus C_1$, $E_1 = (C_1 \parallel T_2)\oplus ID_1$, ${BIF}_1 = h(T_2 \parallel ID_1) \oplus C_2$, $E_3 = h({C}_2 \wedge ID_1) \oplus ID_2$ and $E_4 = h(C_1 \parallel ID_1 \wedge C_2 \parallel ID_2)$

Finally, $D_1$ sends the following message to the cloud server to continue the ownership transfer process: $\langle T_2, E_1, E_3, E_4, {AIF}_1, {BIF}_1 \rangle .$

**Step 3 -** Once the medical server receives the incoming message, it creates a timestamp $T_3$ and checks if the time difference $|T_3 - T_2|$ falls within the acceptable time window $\Delta T$. If this condition is satisfied, the server proceeds to extract the doctor’s identity information. It computes: ${C}_1^* = h(T_3 \wedge CF_1) \oplus AIF_1$, $ID_1^* = h({C}_1 \parallel T_3) \oplus E_2$, $C^*_2 = h(T_3 \parallel ID_1) \oplus BIF_1$ and $ID^*_2 = h(C_2 \wedge ID_1) \oplus E_3$. To validate the authenticity of the message, the server calculates: $E^*_4 = h(C^*_1 \parallel ID_1^* \wedge C^*_2 \parallel ID^*_2)$, and compares $E^*_4$ with the received value $E_4$. If they are equal, the message is deemed authentic. The server then modifies the Access Control List (ACL), revoking doctor $D_1$’s permissions for sensor *n* and granting those rights to doctor $D_2$. As a result, $D_1$ loses access to the corresponding sensor data.

### Password update phase

This subsection describes the procedure for modifying a user’s password. The steps involved are outlined below.

**Step 1 -** The user inserts their smart card and provides $ID^*_{m}$, $B^*_m$ and $PW^*_{m}$. To validate ownership of the smart card, the card executes the following computations:

$$\begin{aligned} AF^*_{m}&= h(ID^*_m \parallel B^*_m \wedge r_{m_1} \parallel PW^*_m),\\ BF^*_m&= h(AF^*_m \wedge r_{m_2}) , \\ CF^*_m&= h(BF^*_m \oplus r_{m_2} \parallel ID^*_m ), \\ DF^*_m&= h(CF^*_m \parallel BF^*_m \wedge r_{m_2}) \end{aligned}$$

Ownership is confirmed by comparing the computed $DF^*_m$ with the stored value $DF_m$ on the smart card.

**Step 2 -** The user inputs $$PW^{**}_m$$ as the new password. The smart card then performs the following computations:

$$\begin{aligned} AF^{**}_m&=h(ID^*_m \parallel B^*_m \wedge r_{m_1} \parallel PW^{**}_m) , \\ BF^{**}_m&= h(AF^{**}_m \wedge r_{m_2}) , \\ CF^{**}_m&= h(BF^{**}_m \oplus r_{m_2} \parallel ID^*_m) , \\ DF^{**}_m&= h(CF^{**}_m \parallel BF^{**}_m \wedge r_{m_2}) \end{aligned}$$

Finally, the value $$DF_m$$ stored in the smart card is updated and replaced with $$DF^{**}_m$$.

### Biometrics update phase

This section outlines the procedure for updating a user’s biometric information. The required steps are detailed below.

**Step 1 -** The user inserts their smart card and provides $ID^*_{m}$, $B^*_m$ and $PW^*_{m}$. To validate ownership of the smart card, the card executes the following computations:

$$\begin{aligned} AF^*_{m}&= h(ID^*_m \parallel B^*_m \wedge r_{m_1} \parallel PW^*_m),\\ BF^*_m&= h(AF^*_m \wedge r_{m_2}) , \\ CF^*_m&= h(BF^*_m \oplus r_{m_2} \parallel ID^*_m ), \\ DF^*_m&= h(CF^*_m \parallel BF^*_m \wedge r_{m_2}) \end{aligned}$$

Ownership is confirmed by comparing the computed $DF^*_m$ with the stored value $DF_m$ on the smart card.

**Step 2 -** The user imprints $$B^{**}_m$$ as the new biometrics. The smart card then performs the following computations:

$$\begin{aligned} AF^{**}_m&=h(ID^*_m \parallel B^{**}_m \wedge r_{m_1} \parallel PW^{*}_m) , \\ BF^{**}_m&= h(AF^{**}_m \wedge r_{m_2}) , \\ CF^{**}_m&= h(BF^{**}_m \oplus r_{m_2} \parallel ID^*_m) , \\ DF^{**}_m&= h(CF^{**}_m \parallel BF^{**}_m \wedge r_{m_2}) \end{aligned}$$

Finally, the value $$DF_m$$ stored in the smart card is updated and replaced with $$DF^{**}_m$$.

## Security analysis

In this section, we formally and informally describe the security of our protocol.

### Formal security analysis

This section provides a comprehensive formal security evaluation of the proposed protocol. We employ established verification techniques such as BAN logic, the Real-Or-Random (ROR) model, and automated verification through the AVISPA tool to rigorously analyse its resilience against a wide range of attacks.

#### Burrows-Abadi-Needham (BAN) logic

In this section, we provide a formal security proof of the proposed protocol using BAN logic. BAN logic is a belief-based framework used to reason about authentication protocols. It allows the derivation of new beliefs from previously held ones. Below, we present the commonly used symbols and inference rules of BAN logic employed in this work.

## BAN logic symbols

- $X \mid \equiv Y$: *X* believes that statement *Y* is true.
- $X \triangleleft Y$: *X* receives a message that contains a statement *Y* from a network agent *Z*.
- $X \mid \sim Y$: *X* has sent a message that contains *Y* to a network agent *Z*.
- $$\#X$$: *X* is fresh.
- $X \leftrightarrow YZ$: *Y* is a secret shared between *X* and *Z*.
- $X \overset{Y}{\leftrightarrow }\ Z$: *Y* is a shared statement between *X* and *Z*.
- $$X \vdash Y$$: *X* can derive *Y*.
- $$X \Rightarrow M_1$$ : *X* controls $$M_1$$

## BAN logic rules

**1. Message Meaning Rule (MMR):**

$$\frac{X \mid \equiv X \mathop {\leftrightarrow }\limits ^{K} Y, \; X \triangleleft \{M_1\}_K}{X \mid \equiv Y \mid \sim M_1}$$

**2. Nonce-Verification Rule (NVR):**

$$\frac{X \mid \equiv \#(M_1), \; X \mid \equiv Y \mid \sim M_1}{X \mid \equiv Y \mid \equiv M_1}$$

**3. Jurisdiction Rule (JR):**

$$\frac{X \mid \equiv Y \Rightarrow M_1, \; X \mid \equiv Y \mid \equiv M_1}{X \mid \equiv M_1}$$

**4. Freshness Rule (FR):**

$$\frac{X \mid \equiv \#(M_1)}{X \mid \equiv \#(M_1, M_2)}$$

**5. Belief Rule (BR):**

$$\frac{X \mid \equiv (M_1, M_2)}{X \mid \equiv M_1}$$

The correctness proof using BAN logic is segmented into five sections as outlined below. In this proof, R represents a reader, and T denotes a tag.

## I. Protocol description

This subsection outlines the communication messages exchanged among the server, the doctor or user, and the sensor or patient.

- $$D \rightarrow M: \{T_1, SD_{2m}, D1_{nj}\}$$
- $$M \rightarrow S: \{SE_n, SN_n, T_2\}$$
- $$S \rightarrow M: \{T_3, SX_n, SD_n\}$$
- $$M \rightarrow D: \{T_4, So_m, Sn_m, SD_{3m}\}$$

## II. Protocol idealization

In this section, we translate the protocol messages into BAN logic notation.

- $$D \rightarrow M: M \triangleleft \{T_1, SD_{2m}, SD_{1m}\}$$
- $$M \rightarrow S: S \triangleleft \{SE_n, SN_n,T_2 \}$$
- $$S \rightarrow M: M \triangleleft \{T_3, SX_n,SD_n \}$$
- $$M \rightarrow D: D \triangleleft \{T_4, So_m,Sn_m, SD_{3m} \}$$

## III. Preliminary assumptions

The initial assumptions considered for the proposed protocol are listed below:

$$\begin{aligned} \text {Assumption 1.}&\quad \text {CS} \mid \equiv (D_m \xleftarrow {\text {KID}_\text {m}} \longrightarrow \text {CS}), \\ \text {Assumption 2.}&\quad \text {CS} \mid \equiv \#(t_1), \\ \text {Assumption 3.}&\quad \text {S} \mid \equiv (\text {CS} \xleftarrow {\text {KID}_\text {m}} \longrightarrow \text {S}), \\ \text {Assumption 4.}&\quad \text {S} \mid \equiv \#(t_2), \\ \text {Assumption 5.}&\quad \text {CS} \mid \equiv (\text {S} \xleftarrow {\text {IDPK}_\text {n}} \longrightarrow \text {CS}), \\ \text {Assumption 6.}&\quad \text {CS} \mid \equiv \#(t_3), \\ \text {Assumption 7.}&\quad D_m \mid \equiv (\text {CS} \xleftarrow {\text {IDPK}_\text {n}} \longrightarrow D_m), \\ \text {Assumption 8.}&\quad D_m \mid \equiv \#(t_4), \\ \text {Assumption 9.}&\quad D_m \mid \equiv \text {S} \Rightarrow (D_m \xleftarrow {\text {CSK}_\text {n}} \longrightarrow \text {S}), \\ \text {Assumption 10.}&\quad \text {S} \mid \equiv D_m \Rightarrow (D_m \xleftarrow {\text {CSK}_\text {n}} \longrightarrow \text {S}). \end{aligned}$$

## IV. Protocol goal

The security goals intended to be achieved by the proposed protocol are as follows:

$$\begin{aligned} \text {Goal 1:}&\quad D_m \mid \equiv D_m \xleftarrow {\text {CSK}_\text {n}} \longrightarrow S, \\ \text {Goal 2:}&\quad S \mid \equiv D_m \xleftarrow {\text {CSK}_\text {n}} \longrightarrow S, \\ \text {Goal 3:}&\quad D_m \mid \equiv S \mid \equiv D_m \xleftarrow {\text {CSK}_\text {n}} \longrightarrow S, \\ \text {Goal 4:}&\quad S \mid \equiv D_m \mid \equiv D_m \xleftarrow {\text {CSK}_\text {n}} \longrightarrow S. \end{aligned}$$

## V. Proof process

Using the rules of BAN logic, the idealised forms of the exchanged messages are analysed to verify whether the protocol meets its intended security goals.

**Step-1:** From Message-1, we derive the following:

$$BL_1: \, \text {CS} \triangleleft ( T_1, SD_{2m}, SD_{1m})$$

**Step-2:** By applying $BL_1$ and Assumption-1 in conjunction with the message meaning rule, we obtain:

$$BL_2: \, \text {CS} \mid \equiv D_m \mid \sim ( T_1, SD_{2m}, SD_{1m})$$

**Step-3:** By employing $BL_2$, along with Assumption-2 and the rule of message freshness, we conclude:

$$BL_3: \, \text {CS} \mid \equiv \#( T_1, SD_{2m}, SD_{1m})$$

**Step-4:** Utilising $BL_2$ and $BL_3$, and invoking the nonce verification rule, we infer the following:

$$BL_4: \, \text {CS} \mid \equiv D_m \mid \equiv ( T_1, SD_{2m}, SD_{1m})$$

**Step-5:** With reference to Message-2, the following expression can be formulated:

$$BL_5: \, \text {S} \triangleleft (T_2, SE_n, SN_n)$$

**Step-6:** Applying $BL_5$ along with Assumption-3 and the message meaning rule, we obtain the following:

$$BL_6: \, \text {S} \mid \equiv \text {CS} \mid \sim (T_2, SE_n, SN_n)$$

**Step-7:** Applying $BL_6$, Using Assumption-4 in combination with the freshness rule, we derive the following:

$$BL_7: \, \text {S} \mid \equiv \#(T_2, SE_n, SN_n)$$

**Step-8:** Applying $BL_6$ and $BL_7$ together with the nonce verification rule, we conclude:

$$BL_8: \, \text {S} \mid \equiv \text {CS} \mid \equiv (T_2, SE_n, SN_n)$$

**Step-9:** Based on Message-3, we express the following:

$$BL_9: \, \text {CS} \triangleleft (T_3, SX_n, SD_n)$$

**Step-10:** By applying $BL_9$ alongside Assumption-5 and the message meaning rule, we derive:

$$BL_{10}: \, \text {CS} \mid \equiv \text {S} \mid \sim (T_3, SX_n, SD_n)$$

**Step-11:** Utilizing $BL_{10}$ together with Assumption-6 and the freshness rule, we infer:

$$BL_{11}: \, \text {CS} \mid \equiv \#(T_3, SX_n, SD_n)$$

**Step-12:** By employing $BL_{10}$ and $BL_{11}$ along with the nonce verification rule, we conclude:

$$BL_{12}: \, \text {CS} \mid \equiv \text {S} \mid \equiv (T_3, SX_n, SD_n)$$

**Step-13:** Referring to Message-4, we obtain:

$$BL_{13}: \, D_m \triangleleft (SD_{3m}, T_4, So_m, Sn_m)$$

**Step-14:** Applying $BL_{13}$ together with Assumption 7 and the message meaning rule, we derive:

$$BL_{14}: \, D_m \mid \equiv \text {CS} \mid \sim (SD_{3m}, T_4, So_m, Sn_m)$$

**Step-15:** By applying $BL_{14}$ in conjunction with Assumption 8 and the freshness rule, we infer:

$$BL_{15}: \, D_m \mid \equiv \#(SD_{3m}, T_4, So_m, Sn_m)$$

**Step-16:** Utilizing $BL_{14}$ and $BL_{15}$ along with the nonce verification rule, we conclude:

$$BL_{16}: \, D_m \mid \equiv \text {CS} \mid \equiv (SD_{3m}, T_4, So_m, Sn_m)$$

**Step-17:** Through the application of $BL_{16}$ alongside the belief rule, we derive:

$$BL_{17}: \, D_m \mid \equiv \text {S} \mid \equiv D_m \xleftarrow {\text {CSK}_\text {n}} \longrightarrow \text {S} (\text {Goal-3})$$

**Step-18:** By applying $BL_{17}$ together with Assumption-9 and the jurisdiction rule, we infer:

$$BL_{18}: \, D_m \mid \equiv D_m \xleftarrow {\text {CSK}_\text {n}} \longrightarrow \text {S} (\text {Goal-1})$$

**Step-19:** With $\text {CSK}_\text {n}$ and using $BL_4$, $BL_8$, $BL_{12}$, and $BL_{16}$, we write:

$$BL_{19}: \, \text {S} \mid \equiv D_m \mid \equiv D_m \xleftarrow {\text {CSK}_\text {n}} \longrightarrow \text {S} (\text {Goal-4})$$

**Step-20:** Applying $BL_{18}$ along with Assumption 10 and the jurisdiction rule, we conclude:

$$BL_{20}: \, \text {S} \mid \equiv D_m \xleftarrow {\text {CSK}_\text {n}} \longrightarrow \text {S} (\text {Goal-2})$$

### Real-or-Random (ROR) model

The formal security of the proposed protocol is analysed using the Real-Or-Random (ROR) model. Within this framework, the entities $I_{DF_m}^{x_1}$, $I_{S}^{x_2}$, and $I_{CS}^{x_3}$ denote the user/doctor, the sensor node, and the medical server, respectively, where $x_1$, $x_2$, and $x_3$ represent specific instances of these roles. The ROR model is grounded in the well-established Dolev-Yao (DY) model, under which the adversary $\mathcal {A}$ has complete control over the communication channels. Consequently, $\mathcal {A}$ is capable of performing a range of actions, such as intercepting, modifying, blocking, forging, or deleting messages, by issuing the following types of queries during the communication between $D_{m}$, *CS*, and *S*:

- **Execute** $I^{x_1}_{D_m}$, $I^{x_2}_{S}$: This query allows $\mathcal {A}$ to intercept messages exchanged between $I^{x_1}_{D_m}$ and $I^{x_2}_{S}$ over an insecure channel.
- **Corruptdevice:** This query enables $\mathcal {A}$ to extract stored information from the databases of $I^{x_1}_{D_m}$ or $I^{x_2}_{S}$.
- **Send** $( {I^x}, \text {message})$: This query allows $\mathcal {A}$ to transmit a message to additional participants, who then respond to $\mathcal {A}$.
- **Reveal** $( {I^x} )$: This query grants $\mathcal {A}$ the authorization to access the current session key.
- **Test** $( {I^x} )$: This query either returns the session key $S$ or a random value, depending on the outcome of the coin flip, resulting in $b = 0$ or $b = 1$.

**Partnering:** Two instances $I^{x_1}$ and $I^{x_2}$ are considered partners if:

1. Both $I^{x_1}$ and $I^{x_2}$ are in acceptable states.
2. They mutually authenticate each other and share the same session ID.
3. They are mutually exclusive.

#### Theorem 1

$\text {Adv}_{s}(t)$ is the chance of breaking the session key security of the proposed work in polynomial time *t*. The variables $q_h$, $q_s$, $|\text {hash}|$, *l*, and $D_p$, respectively, represent the number of hash queries, send queries, range space of the hash function, biometric bit information, and password dictionary size. Consequently, the following is the conclusion:

$$\text {Adv}_s(t) \le \frac{q_h^2}{|\text {hash}|} + \frac{q_s}{2^{l-1} \cdot |D_p|} + \text {Adv}_{\text {rand}}(t).$$

**Introduction to game sequence -** To formally analyse the session key security of the proposed protocol, we adopt a game-based proof technique under the Real-or-Random (ROR) security model. In this approach, a sequence of games is defined, beginning with the real attack scenario and gradually transitioning to an idealised experiment. Each game differs from the previous one by a small and well-controlled modification, such as restricting the adversary’s capabilities or replacing certain protocol components with idealised counterparts. The core idea behind this methodology is that if the adversary’s success probability changes only negligibly between any two consecutive games, then the adversary’s overall advantage in the real protocol execution can be tightly bounded. The difference in the adversary’s success probability between successive games is quantified using standard cryptographic assumptions, including the security of the hash function, the unpredictability of random nonces, and the resistance to offline password and biometric guessing attacks. Finally, by applying the triangle inequality across all game transitions, the adversary’s total advantage in distinguishing the real session key from a random value is upper-bounded by the sum of these individual differences, thereby establishing the session key security of the proposed protocol.

**Game G0:-** In this game, we simulate the actual attack scenario. The adversary interacts with honest parties and uses the Test query to distinguish the real session key from a random one. We define:

$$\text {Adv}_s(t) = \left| 2 \cdot \Pr [\text {Succ}_{G_0}] - 1 \right|$$

**Game G1:-** In this game, the adversary uses the Execute query to eavesdrop on messages $M_1, M_2, M_3, M_4$. However, due to the secrecy of $\text {PID}_n$, $r_{m_5}$, and $r_{m_6}$, $\mathcal {A}$ cannot compute $\text {IDPK}_n$. Thus,

$$\Pr [\text {Succ}_{G_0}] = \Pr [\text {Succ}_{G_1}]$$

**Game G2:-** In Game $G_2$, the adversary uses Send and Hash queries to impersonate legitimate users or generate valid messages. However, to forge a valid session key, $\mathcal {A}$ must guess the values of $\text {PID}_n$, $r_{m_5}$, and $r_{m_6}$. As these are protected and not transmitted explicitly, and $h(\cdot )$ is modelled as a random oracle, the success advantage is bounded by:

$$|\Pr [\text {Succ}_{G_2}] - \Pr [\text {Succ}_{G_1}]| \le \frac{q_h^2}{2 \cdot |\text {hash}|}$$

**Game G3:-** Here, $\mathcal {A}$ corrupts the smart card or user/sensor device to extract stored values. They attempt offline dictionary and biometric guessing attacks using the stored values. Since password and biometric data are masked using random numbers and a secure hash function, the probability of successful guessing is:

$$|\Pr [\text {Succ}_{G_3}] - \Pr [\text {Succ}_{G_2}]| \le \frac{q_s}{2^l \cdot |D_p|}$$

**Game G4:-** In this final game, $\mathcal {A}$ attempts to directly guess the session key:

$$\text {IDPK}_n = h(\text {PID}_n \wedge r_{m_5} \parallel r_{m_6})$$

Without knowing the inputs to $h(\cdot )$, and since it is modelled as a random oracle, the adversary’s chance of success is no better than random guessing:

$$\Pr [\text {Succ}_{G_4}] = \frac{1}{2}.$$

Now, from the definition of advantage in the ROR model, we know:

$$\frac{1}{2} \text {Adv}_s(t) = \left| \Pr _s[\text {Succ}_0] - \frac{1}{2} \right| = \left| \Pr _s[\text {Succ}_1] - \frac{1}{2} \right| = \left| \Pr _s[\text {Succ}_1] - \Pr _s[\text {Succ}_4] \right|$$

Now we expand $\left| \Pr _s[\text {Succ}_1] - \Pr _s[\text {Succ}_4] \right|$ using the triangle inequality:

$$\left| \Pr _s[\text {Succ}_1] - \Pr _s[\text {Succ}_4] \right| \le \left| \Pr _s[\text {Succ}_1] - \Pr _s[\text {Succ}_2] \right| + \left| \Pr _s[\text {Succ}_2] - \Pr _s[\text {Succ}_3] \right| + \left| \Pr _s[\text {Succ}_3] - \Pr _s[\text {Succ}_4] \right|$$

Using the bounds derived from each game transition:

- From Game 1 and Game 2:
  $$\left| \Pr _s[\text {Succ}_1] - \Pr _s[\text {Succ}_2] \right| \le \frac{q_h^2}{2|\text {hash}|}$$
- From Game 2 and Game 3:
  $$\left| \Pr _s[\text {Succ}_2] - \Pr _s[\text {Succ}_3] \right| \le \frac{q_s}{2^l |D_p|}$$
- From Game 3 and Game 4:
  $$\left| \Pr _s[\text {Succ}_3] - \Pr _s[\text {Succ}_4] \right| \le \frac{1}{2}\text {Adv}_{\text {rand}}(t)$$

Combining all three bounds, we get:

$$\left| \Pr _s[\text {Succ}_1] - \Pr _s[\text {Succ}_4] \right| \le \frac{q_h^2}{2|\text {hash}|} + \frac{q_s}{2^l |D_p|} + \frac{1}{2}\text {Adv}_{\text {rand}}(t).$$

Multiplying both sides by 2, we get:

$$\text {Adv}_s(t) \le \frac{q_h^2}{|\text {hash}|} + \frac{2q_s}{2^l |D_p|} + \text {Adv}_{\text {rand}}(t)$$

Simplifying:

$$\text {Adv}_s(t) \le \frac{q_h^2}{|\text {hash}|} + \frac{q_s}{2^{l-1} |D_p|} + \text {Adv}_{\text {rand}}(t)$$

Therefore, the adversary’s advantage in breaking the session key remains bounded and negligible under the hardness assumptions and secure randomness used in the protocol.

### Formal security analysis by AVISPA tool

In this section, the security of the CSMAE protocol is formally evaluated with the aid of the AVISPA (Automated Validation of Internet Security Protocols and Applications) framework. AVISPA is a well-established platform for analysing authentication protocols and is frequently utilised to examine their robustness against threats such as replay attacks and man-in-the-middle (MITM) attacks. The framework incorporates four distinct back-end engines for conducting the analysis:

1. **OFMC (On-the-Fly Model Checker):** Performs dynamic exploration of the protocol’s state space to identify potential vulnerabilities.
2. **CL-AtSe (Constraint-Logic-based Attack Searcher):** Utilises constraint-solving methods to systematically search for possible protocol attacks.
3. **SATMC (SAT-based Model Checker):** Translates the verification problem into a Boolean satisfiability problem, enabling efficient detection of flaws.
4. **TA4SP (Tree Automata-based on Automatic Approximations for the Analysis of Security Protocols):** Employs tree automata techniques for approximating the protocol’s behaviour to uncover weaknesses.

Comprehensive explanations of these verification back-ends are provided in the official AVISPA documentation^40^. To model and test security protocols, AVISPA employs the High-Level Protocol Specification Language (HLPSL), a role-oriented language. Within HLPSL, two types of roles are defined: basic roles, which specify the behaviour of individual protocol participants, and composition roles, which capture the various ways these participants interact.

To represent adversarial actions, HLPSL treats the intruder (denoted as i) as if it were a legitimate participant, thereby simulating malicious behaviour. The HLPSL specification is first converted into an Intermediate Format (IF) using the HLPSL2IF translator. This IF is then processed by one of the AVISPA back-end engines, which produces the Output Format (OF) containing the final security analysis results.

## Examination of simulation outcomes

The CSMAE protocol has been evaluated using the well-established back-ends, namely OFMC and CL-AtSe, integrated within SPAN (Security Protocol Animator), which serves as a graphical front-end for the AVISPA framework^40^. For a comprehensive assessment of CSMAE, three key verification dimensions are considered:

1. **Executability Verification:** Confirms that the protocol is capable of reaching a state in which potential attacks could manifest during its execution.
2. **Replay Attack Resistance:** Validates the protocol’s ability to withstand adversarial attempts based on replaying previously transmitted messages.
3. **Dolev-Yao Model Evaluation:** Investigates the protocol’s security under the classical Dolev-Yao adversarial model, which assumes an attacker with complete control over public communication channels.

In the following GitHub repository https://github.com/divyanshugairola2023-cell/csmae-avispa/blob/main/csmae.txt, we have properly encoded the CSMAE protocol in HLPSL (High-Level Protocol Specification Language), with specifications that are non-trivial in nature. The executability assessment validates that the protocol is capable of reaching states where potential adversarial actions could arise, thereby meeting its design requirements. In addition, the protocol has been simulated to evaluate bounded session executions.

For replay attack verification, the OFMC and CL-AtSe back-ends examine whether honest participants are able to successfully execute the protocol even in the presence of a passive adversary. These back-ends further analyse the system’s robustness against man-in-the-middle threats under the Dolev-Yao attack model, thereby ensuring a thorough security evaluation.

**Fig. 6:**
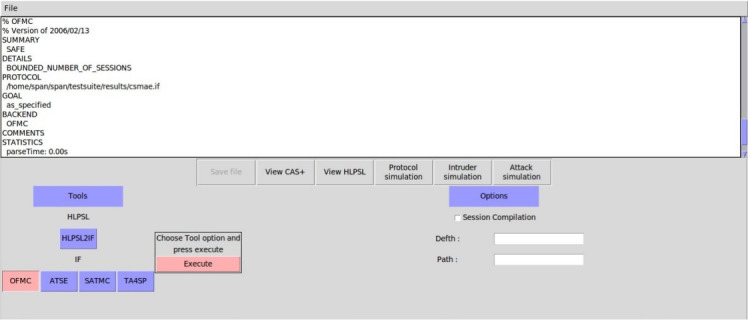
AVISPA OFMC result.

**Fig. 7:**
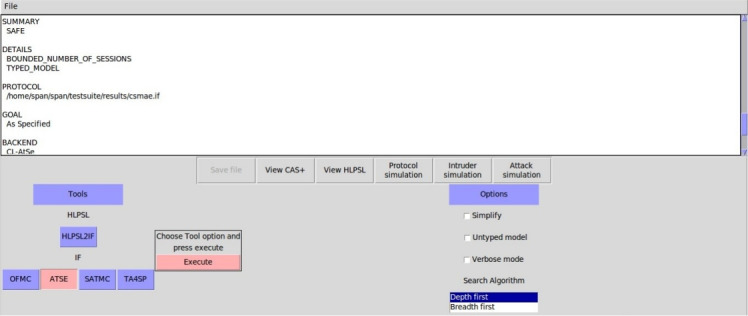
AVISPA ATSE result.

The simulation findings, as depicted in Figs. 6 and 7, demonstrate that all major verification goals have been fulfilled by CSMAE. These include executability testing of the HLPSL specifications, detection of replay-based intrusions, and validation under the Dolev-Yao model. As a result, the protocol proves to be secure against both replay and man-in-the-middle attacks.

### Informal security analysis

This section presents an informal assessment of the security attributes of our protocol.

#### Replay attack

In the CSMAE protocol, the server employs timestamp validation to mitigate replay attacks. For example, upon receiving the message $$\langle T_1, SD_{2m}, SD_{1m} \rangle$$, the server verifies whether $$|T_2 - T_1| \le \Delta T$$. If this condition fails, the session is aborted. An adversary may attempt to circumvent this check by substituting $$T_1$$ with a recent timestamp $$T^{**}_1$$ and transmitting $$\langle T^{**}_1, SD_{2m}, SD_{1m} \rangle$$ to the server. To counter this, the server computes

$$r_{m4} = h(DF_m \wedge CF_m \parallel T^{**}_1) \oplus SD_{1m},$$

$$SD^{**}_{2m} = h(DF_m \wedge SD_{1m} \parallel r_{m4}) \oplus PID_n,$$

and then checks whether $$SD^{**}_{2m}$$ equals the received $$SD_{2m}$$. A mismatch indicates that the timestamp has been altered.

A similar defence applies in Step 3 of the authentication phase for the message $$\langle T_3, SX_n, SD_n \rangle$$. An attacker may replace $$T_3$$ with a falsified value $$T^{**}_3$$ and forward $$\langle T^{**}_3, SX_n, SD_n \rangle$$. The server first ensures that $$|T_4 - T_3| \le \Delta T$$, then computes

$$SD^{*}_n = h(T^{**}_3 \wedge IDPK_n \parallel SF_n) ,$$

and compares it with the received $SD_n$. If the values do not match, the server concludes that the timestamp has been manipulated.

#### Known-session-specific temporary information attack

For resistance against a known-session-specific temporary information attack, the protocol must ensure that the adversary cannot reconstruct the session key ($IDPK_n$) even if the session-related random values ($r_{m4}$, $r_{m5}$, and $r_{m6}$) are exposed. In the registration stage, the server’s memory is assumed to be tamper-resistant, preventing unauthorised access to the identity of the $n^{th}$ sensor ($PID_n$) stored in its database. As a result, even if an attacker manages to obtain the random numbers $r_{m5}$ and $r_{m6}$, they are still unable to compute $IDPK_n = h( PID_n \wedge r_{m5} \parallel r_{m6})$.

#### Perfect forward secrecy

A protocol is considered to achieve perfect forward secrecy if, even when an adversary gains knowledge of the long-term credentials (such as identities or secret keys), the session key $IDPK_n$ remains inaccessible. In the case of the CSMAE scheme, the session key is constructed as $IDPK_n = h( PID_n \wedge r_{m5} \parallel r_{m6})$, which incorporates fresh random values $r_{m5}$ and $r_{m6}$. Consequently, disclosure of the server’s secret key or sensor passwords does not enable the attacker to reconstruct $IDPK_n$.

#### Resistance to insider attack

As described in section v, during the registration stage, the request message forwarded to the server is $\langle AF_m, ID_m \rangle$ rather than transmitting the password directly. Hence, an insider adversary cannot recover the password $PW_m$ from this communication. Specifically, $AF_m$ is defined as $h(ID_m\parallel B_m \wedge r_{m1} \parallel PW_m)$, which incorporates the random value $r_{m1}$, thereby preventing the extraction of $PW_m$.

#### Known-key secrecy

In the proposed scheme, the session key $IDPK_n = h( PID_n \wedge r_{m5} \parallel r_{m6})$ is derived using session-dependent random values $r_{m5}$ and $r_{m6}$, which are freshly generated for every execution. As a result, disclosure of a past session key does not affect the secrecy of keys established in other sessions. This property ensures that the protocol achieves known-key secrecy.

#### Physician impersonation attack

For an adversary to successfully impersonate a legitimate physician, they may attempt to transmit a seemingly valid message $\langle T_1, SD_{2m}, SD_{1m} \rangle$ to the server. Upon receiving this, the server independently computes $r^{*}_{m4} = h(DF_m \wedge CF_m \parallel T_1) \oplus SD_{1m}$, followed by $PID^{*}_n = h(DF_m \wedge SD_{1m} \parallel r^{*}_{m4}) \oplus SD_{2m}$, and then derives $SD^{*}_{2m} = h(DF_m \wedge SD_{1m} \parallel r^{*}_{m4}) \oplus PID^{*}_n$. Since these values are generated using parameters securely held by the server, rather than extracted from the adversary’s message, the server finally checks whether $SD^{*}_{2m}$ matches $SD_{2m}$. If this verification fails, the request is rejected, ensuring that the protocol effectively resists physician impersonation attempts.

#### Server impersonation attack

For an adversary to masquerade as the server, they may attempt to send a seemingly valid message $\langle SE_n, SN_n, T_2 \rangle$ to the sensor. Upon receiving this, the sensor locally computes $N^{*}_k$ using its securely stored parameters (rather than those included in the received message) and compares the result with $SN_n$. If the two values do not match, the sensor concludes that the message is not authentic, thereby preventing server impersonation.

Similarly, an attacker might try to deceive the user by transmitting a semi-valid message $\langle T_4, SO_m, Sn_m, SD_{3m} \rangle$. In this case, the user independently computes $SD^{*}_{3m} = h(AF_m \wedge SO_m \parallel Sn_m \parallel T_4)$ and checks whether it equals the received $SD_{3m}$. Although $SO_m$, $Sn_m$, and $T_4$ are derived from the adversary’s fabricated message, the value $AF_m$ is exclusively known to the user. Hence, the equality test fails for a forged message, ensuring that the proposed protocol resists server impersonation attempts.

#### Sensor impersonation attack

In an attempt to impersonate a sensor, an adversary may transmit a semi-valid message $\langle T_3, SX_n, SD_n \rangle$ to the server. Upon receiving it, the server generates $CSK_n = h( PID_n \wedge r_{m5} \parallel r_{m6})$ using its own securely stored parameters $r_{m5}$, $PID_n$, and $r_{m6}$, which are independent of the attacker’s message. The server then computes $SD^{*}_n = h(T_3 \wedge CSK_n \parallel SF_n)$ and compares it with the received $SD_n$. If the message originates from an illegitimate source, the comparison fails, confirming that the sender is not a genuine sensor. Thus, the proposed protocol effectively prevents sensor impersonation attempts.

#### Password guessing attack

A scheme is resilient to password-guessing attacks when an adversary is unable to derive the user’s password from any publicly transmitted information. In the CSMAE protocol, the values $AF_m$ and $ID_m$ are securely communicated during the registration process, ensuring that an attacker cannot directly exploit them. Moreover, even if these parameters are exposed, the password $PW_m$ cannot be obtained since it is combined with the random nonce $r_{m1}$, which remains unknown to the adversary. Consequently, the protocol effectively safeguards against password-guessing attempts.

#### Denial of service attack

In the proposed protocol during the login and authentication phase, for each session, we have used five time stamps, $T_1, T_2, T_3$, $T_4$, and $T_5$, to ensure the freshness of transmitted messages. Furthermore, in each session we used random numbers $r_{m_4}$, $r_{m_5}$, and $r_{m_6}$ to prevent adversaries from replaying previously sent messages multiple times. So if the adversary tries to overwhelm the server and send an old message $\langle T_1, SD_{2m}, SD_{1m} \rangle$ multiple times, the server will block this request immediately by recording the current timestamp $T_2$ and verifying the freshness of the request by checking whether $| T_2-T_1 | < \Delta T$. Additionally, the server will calculate $SD_{2m}^{*} = h(DF_{m} \wedge SD_{1m} \parallel r_{m_4}^{*}) \oplus PID_n^{*}$ and verify whether the calculated $SD_{2m}^{*}$ is equal to the received $SD_{2m}$. Hence, the scheme is capable of resisting denial-of-service (DoS) attacks by effectively detecting and blocking repeated transmission attempts.

#### Stolen verifier attack

A protocol achieves resistance to a stolen verifier attack if an adversary cannot reconstruct the session key by compromising the verifier-related information stored in memory. In the proposed scheme, the memory is assumed to be tamper-resistant. Even in a scenario where this assumption does not hold, the adversary still cannot derive the session key since it relies on the random nonce $r_{m5}$ and $r_{m_6}$, which remain unknown to the attacker.

#### Key compromise impersonation (KCI) attack

A scheme is secure against a KCI attack if an adversary, despite obtaining the server’s long-term secret key, cannot impersonate a legitimate entity and establish a session key with another participant. In the proposed protocol, the session key $IDPK_n = h( PID_n \wedge r_{m5} \parallel r_{m6})$ incorporates the random nonces $r_{m5}$ and $r_{m6}$, which are unknown to the adversary. Consequently, even with knowledge of the server’s secret key, the attacker cannot compute $IDPK_n$, ensuring protection against KCI attacks.

#### Man-in-the-middle attack

The proposed protocol defends against man-in-the-middle attacks by enforcing mutual authentication between the communicating parties before the session key is established. As a result, even if an adversary intercepts the communication and gains access to the transmitted parameters, they cannot successfully impersonate either participant in the exchange.

We performed a security comparison with existing related protocols, as illustrated in the Table 4. The results highlight that the proposed scheme effectively counters all prevalent attacks in e-healthcare systems and integrates all the contemporary security properties.

**Table 4:** Various attacks and features comparison.

Attacks/Features	^41^	^3^	^2^	^10^	^42^	^43^	^44^	^11^	^32^	CSMAE
Replay Attack	−	$\checkmark$	$\checkmark$	$\checkmark$	$\checkmark$	−	$\times$	$\checkmark$	$\times$	$\checkmark$
Known-Session-Specific Temporary Information Attack	$\checkmark$	−	−	−	−	−	−	−	−	$\checkmark$
Perfect Forward Secrecy	−	−	−	−	−	−	−	−	−	$\checkmark$
Insider Attack	−	$\checkmark$	$\checkmark$	$\times$	$\times$	−	$\times$	$\checkmark$	$\checkmark$	$\checkmark$
Known Key Secrecy	$\checkmark$	$\checkmark$	−	$\checkmark$	$\checkmark$	−	$\times$	$\times$	$\checkmark$	$\checkmark$
User Impersonation Attack	$\checkmark$	$\times$	−	$\checkmark$	$\times$	−	$\checkmark$	$\checkmark$	$\checkmark$	$\checkmark$
Server Impersonation Attack	$\checkmark$	$\times$	−	−	−	−	−	−	−	$\checkmark$
Sensor Impersonation Attack	$\checkmark$	$\times$	−	−	−	−	−	−	−	$\checkmark$
Password Guessing Attack	−	$\checkmark$	−	$\checkmark$	$\checkmark$	−	$\checkmark$	$\times$	$\checkmark$	$\checkmark$
Denial Of Service Attack	−	$\checkmark$	$\checkmark$	$\times$	$\checkmark$	−	$\checkmark$	$\times$	$\checkmark$	$\checkmark$
Stolen Verifier Attack	−	−	−	−	−	−	−	−	−	$\checkmark$
Key Compromise Impersonation	−	$\checkmark$	$\checkmark$	$\checkmark$	$\checkmark$	−	$\times$	$\times$	$\checkmark$	$\checkmark$
Man-In-The-Middle Attack	$\checkmark$	$\checkmark$	$\checkmark$	$\checkmark$	$\checkmark$	−	$\checkmark$	$\checkmark$	$\times$	$\checkmark$
Password Update	$\times$	$\times$	$\times$	$\times$	$\checkmark$	$\times$	$\checkmark$	$\times$	$\times$	$\checkmark$
Ownership Transfer	$\times$	$\times$	$\times$	$\times$	$\times$	$\times$	$\times$	$\times$	$\times$	$\checkmark$

$\times$: The protocol is susceptible to that attack or doesn’t have that security feature..

$\checkmark$: The protocol is secure against that attack or has that security feature..

−: Not mentioned.

## Performance analysis

This section presents the real-time implementation of the proposed CSMAE scheme, followed by an evaluation of its computational and communication overheads, along with a description of the experimental setup.

### Real time implementation

**Fig. 8:**
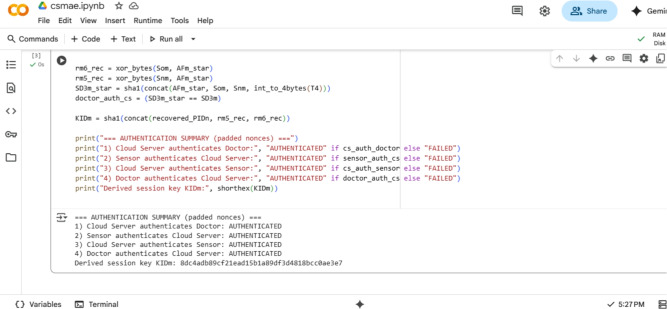
Python code simulation result.

A PC-based simulation of the proposed protocol was carried out using Python to examine the data flow, authentication procedure, and timing performance, as summarised in the Table 5 and depicted in Fig. 8. The findings validate that mutual authentication is successfully achieved among all involved entities, namely the Doctor, Server, and Sensor.

The simulation was implemented on a 64-bit Windows 10 machine equipped with 4 GB RAM, an AMD Ryzen 3-2200u processor (2.50 GHz), and a Radeon Vega 2 graphics card (2 GB). Within this environment, the protocol demonstrated secure and efficient performance.

The implementation relied on lightweight cryptographic primitives, including a one-way hash function, bitwise AND, XOR, and concatenation operations. These primitives are well-suited for deployment on resource-constrained devices such as the ESP32 microcontroller, which is widely adopted in real-time medical IoT applications due to its efficiency and suitability for constrained environments.

**Fig. 9:**
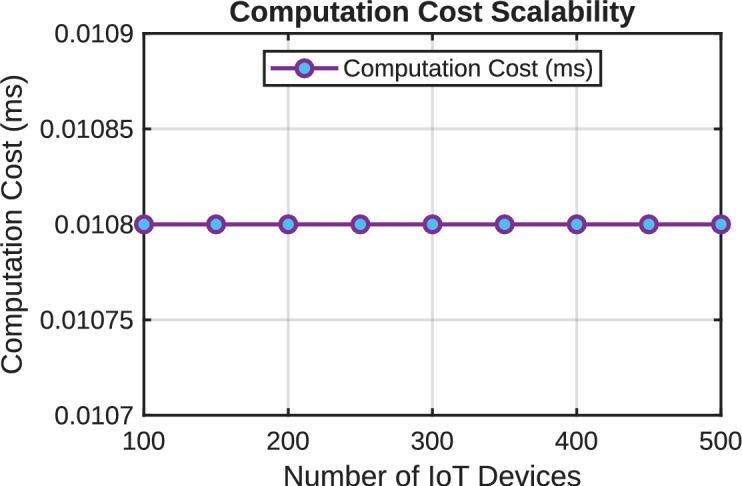
Computation cost scalability analysis.

**Fig. 10:**
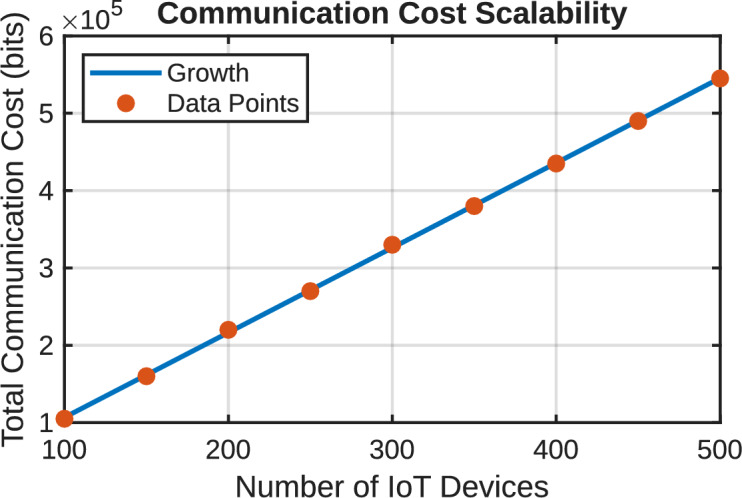
Communication cost scalability analysis.

In addition, a scalability analysis of the protocol was conducted using Python. The evaluation reveals that the computational cost remains stable, irrespective of the increase in the number of IoT devices. In contrast, the communication overhead grows linearly with the number of devices. This performance behaviour is illustrated in the Figs. 9 and 10.

**Table 5:** Computed protocol parameters: normal values and hash values.

Parameter	Normal value(hex)	Hash value (hex)
$ID_m$	95640afda4a4b3a3165128c99e172488525edfc7	a34d5e8f0ccb6594d7187843fead5aeffebcd39d
$PW_m$	fbc67bcd5b74e638a832ac1a142b8a7fe1ad7d4a	415f738110e064af26ded3f570eb84246773437d
$B_m$	f0f01e0b463c48f3d17555af2a82eff102d6166a	be65cf67f7ae308d61aa6af9d3e906b0f63fe827
$r_{m1}$	01020304	12dada1fff4d4787ade3333147202c3b443e376f
$r_{m2}$	05060708	85d9651d9a399a67e015d047fd9e6a941e6b20bb
$r_{m3}$	475a82ec	30772a3f4f6451f1abc816b5627e605a924f6317
$r_{m4}$	090a0b0c	3c6e2fdd37a5ca7f462565587d16c186e4cb5ebd
$SD_{1m}$	03ba2eb6177798e97fb0c7739af58b4dd7e48bea	18bdd1cfa62134ec5649f3bfe228bc5d44934c8e
$SD_{2m}$	ef5d4f8e6813693b448ad1124415ef79dfcddfd8	1b7701f13fe7d4d7fd146ebe2f4069f8b1bdd399
$SE_n$	c63bea86c547b094f87e42f71509021d74d85408	be16b040d35150065be5d49ee57f996ef68ac4cf
$SN_n$	b792dd7c91b32cec802e68ec96ff5f26c306663a	97d897382138771d40fd381da2c473548438457d
$r_{m5}$	0d0e0f10	1f6af60d784769e69886817aacadc24ed56a11cc
$r_{m6}$	11121314	47e78f65cec71555114ad348ed78722cbf2effa4
$SX_n$	fc36f6f6c3dbe632188f32e993901ce43bf9e9b4	821e30ec8764620da3e6d5ee9f02ae29bddbfe0c
$SD_n$	2b7ca646a5b927e8e488653582ec1d741a5bd002	f18af5635aabcf73096e0ed7da522fe1e930c76c
$SD_{3m}$	889df31dd70028467c5432db19988fad989ed535	da573f74ed8cdcc8345efbeaa7125534f9a22243
$So_m$	618cd476871a66b970ca6f94da72f87cabe70827	67daf7743eea796324ff9384bcc4a13a59aff416
$Sn_m$	7d90c872871a66b970ca6f94da72f87cabe70827	e7bef1cb67a801c8c9c83de6d3a779ade0126011

### Computation cost

In this part, we analyse the computational efficiency of the proposed CSMAE protocol by comparing it with several existing approaches. The Table 6 summarises the overall computational overhead of our scheme alongside related protocols. The execution times of the cryptographic primitives are taken from^45^. Based on this evaluation, the approximate processing times are as follows: a one-way hash function requires $0.0004 \, ms$, symmetric encryption or decryption consumes $0.1303 \, ms$, elliptic curve scalar multiplication takes $7.3529 \, ms$, the biometric hash function requires $0.01 \, ms$, and the cryptographically secure pseudo-random number generator (PRNG) operates in $0.0004 \, ms$.

Kestha et al.’s scheme^2^ utilised 5 multiplication operations, 8 addition operations and 10 hash functions. Mohit et al.’s protocol^41^ used 6 signature operations, 9 symmetric encryption/decryption operations and 35 hash functions. Li et al.’s^43^ protocol involved 7 signature operations, 15 symmetric encryption/decryption operations and 36 hash functions. Sahoo et al.’s^42^ scheme utilised 15 hash functions, 6 symmetric encryption/decryption operations and 7 elliptic curve addition/multiplication operations. Zhou et al.’s^44^ protocol used 36 hash functions. Alzahrani et al.’s^3^ scheme involved 18 hash functions, 6 random number generators, 12 elliptic curve addition operations and 12 elliptic curve multiplication operations. Chandrakar et al.’s^10^ protocol utilised 10 signature operations, 18 symmetric encryption/decryption operations and 59 hash functions. Deebak et al.’s scheme^11^ used 8 signature operations, 17 symmetric encryption/decryption operations, 3 modular exponent operations and 45 hash functions.

**Fig. 11:**
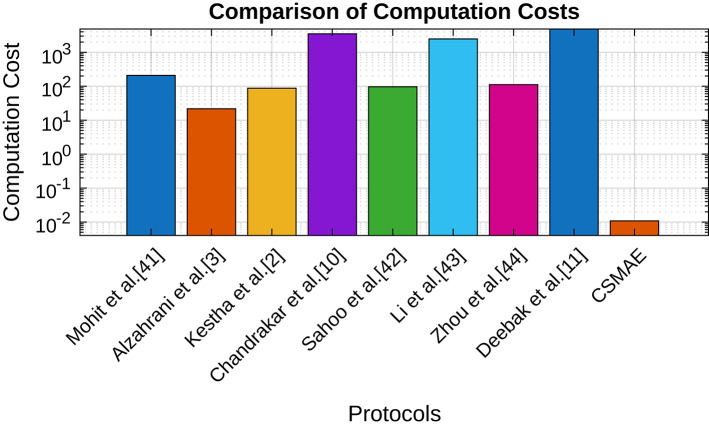
Computation cost comparison.

In comparison, the proposed protocol does not rely on operations such as elliptic-curve multiplication, modular arithmetic, CRC, permutations, or encryption/decryption. Instead, it employs only 18 hash functions and three random-number generators. Consequently, the proposed protocol achieves a lower computational overhead than existing schemes, as depicted in Fig. 11.

**Table 6:** Computation Cost Comparison.

Protocols	Computation costs (In milliseconds)
Mohit et al.^41^	208.6
Alzahrani et al.^3^	21.702
Kestha et al.^2^	87.44
Chandrakar et al.^10^	3503
Sahoo et al.^42^	96.34
Li et al.^43^	2470.44
Zhou et al.^44^	111.35
Deebak et al.^11^	12000
CSMAE	0.0108

### Communication cost

**Fig. 12:**
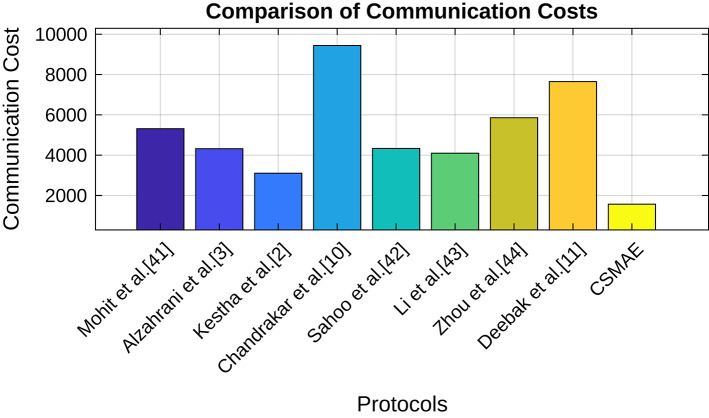
Communication cost comparison.

This section presents a comparison of the communication overhead of the proposed scheme against existing protocols, as summarised in Table 7 and graphically depicted in Fig. 12. In our protocol, we have exchanged four messages for communication between the doctor, cloud server, and patient. The communication requirements for different parameters are calculated as follows: 160 bits are allocated for identity or password information, 32 bits are required for timestamps, 120 bits are used for auxiliary parameters, 160 bits are assigned for the hash function and 32 bits for random nonces (Table 7).

In the proposed protocol, communication during the login phase involves the exchange of $\{T_{1}, SD_{2m}, SD_{1m}\}$, whereas the authentication phase transmits $\{SE_{n}, SN_{n}, T_{2}\}$, $\{T_{3}, SX_{n}, SD_{n}\}$ and $\{T_{4}, Sn_{m}, SO_{m}, SD_{3m}\}$. The bit-level communication overhead for the login phase $\{T_{1}, SD_{2m}, SD_{1m}\}$ amounts to $32 + 160 + 160 = 352$ bits, while the first message of the authentication phase $\{SE_{n}, SN_{n}, T_{2}\}$ incurs $32 + 160 + 160 = 352$ bits. The second message $\{T_{3}, SX_{n}, SD_{n}\}$ amounts to $32 + 160 + 160 = 352$ bits, and the final message $\{T_{4}, Sn_{m}, SO_{m}, SD_{3m}\}$ amounts to a cost of $32 + 160 + 160 + 160 = 512$ bits. Thus, the cumulative communication requirement for both phases equals 1568 bits.

**Table 7:** Communication cost comparison.

Protocols	Communication costs (In bits)
Mohit et al.^41^	5312
Alzahrani et al.^3^	4320
Kestha et al.^2^	3104
Chandrakar et al.^10^	9440
Sahoo et al.^42^	4332
Li et al.^43^	4096
Zhou et al.^44^	5856
Deebak et al.^11^	7648
CSMAE	1568

### Energy consumption

When the proposed key agreement protocol is executed, the participating devices expend battery energy while deriving the session secret key. For a wireless communication setting, the energy consumed to compute the session key on devices such as a Raspberry Pi, a laptop, and a mobile phone can be estimated using the relation $E_t = C_p \times C_q$.

The computational cost of the proposed protocol is $C_p = 0.0108\,\text {ms}$ and the CPU (Ryzen 3 2200U) peak power consumption is constant at $C_q = 15\,\text {W}$ (Nominal TDP), the resulting energy usage becomes $E_t = 0.0000108\,\text {s} \times 15\,\text {W}= 0.000162 J =0.162 mJ$ per run. Hence, executing the proposed key agreement protocol for security authentication among all participating entities in the cloud-computing environment, while establishing the session secret key, consumes about $0.000162 J$ of energy. The Table 8 summarises the overall energy consumption overhead of our protocol with other existing protocols. It clearly shows our scheme is using very less energy as compared to other protocols.

**Table 8:** Energy consumption comparison.

Protocols	Energy consumption in joules
Mohit et al.^41^	3.129
Alzahrani et al.^3^	0.32553
Kestha et al.^2^	1.3116
Chandrakar et al.^10^	52.545
Sahoo et al.^42^	1.4451
Li et al.^43^	37.0566
Zhou et al.^44^	1.67025
Deebak et al.^11^	180
CSMAE	0.000162

### Limitations

While the proposed scheme is lightweight and satisfies the targeted security objectives under the stated assumptions, it has not been evaluated against a post-quantum threat model. Consequently, its security and performance guarantees may not carry over to a post-quantum setting, where adversaries are assumed to possess quantum computational capabilities and where alternative design choices such as the adoption of post-quantum cryptographic primitives may be necessary. As future work, we intend to investigate post-quantum-secure variants of the protocol and to conduct formal security analysis in the presence of quantum-capable adversaries.

## Conclusion

The presented paper introduces an architecture of a cloud-based key agreement scheme designed for secure communication in e-healthcare systems using simple cryptographic functions as AND, XOR, concatenate, and SHA-1 hashing function. The presented solution allows for mutual authentication between parties and ensures the legitimate involvement of all parties in the cloud environment. The main novelty of this research lies in its comprehensive testing approach, which includes formal methods (BAN logic, RoR, AVISPA) and simulation techniques (Python), combined with practical informal security considerations using application specific examples. The performance of the proposed scheme was evaluated based on computational complexity, communication overhead, and scalability aspects. Overall, results show that the proposed architecture is lightweight, resistant to common types of attacks, and satisfies essential security requirements. In addition, comparative studies are conducted to show that the presented design outperforms other existing designs in terms of communication and computational complexities, making it suitable for real world deployment. Finally, future directions will include the extension of the healthcare related security context of the proposed architecture to integrate blockchain technologies and post quantum cryptography architectures, illustrated through a practical demonstration of their integration.

## Data Availability

The data supporting the findings of this study are available in the GitHub repository “divyanshugairola2023- cell”, accessible through the link [https://github.com/divyanshugairola2023-cell/csmae-avispa/blob/main/csmae.txt]. The repository contains the HLPSL (High-Level Protocol Specification Language) encoding of the proposed CSMAE protocol, including non-trivial specifications used for formal verification and analysis.
